# Chemically Programmable Underwater Sound‐Absorbing Metamaterial via MXene Self‐Assembly

**DOI:** 10.1002/advs.76139

**Published:** 2026-06-15

**Authors:** Ziwen Gan, Ranran Qi, Mingyi Liao, Bowen Chen, Chen Cheng, Wei Tu

**Affiliations:** ^1^ College of Transportation Engineering Dalian Maritime University Dalian Liaoning China

**Keywords:** control logic, fabrication, layered structure, materials science, metamaterial, nanotechnology, optoelectronics

## Abstract

Research on underwater acoustic metamaterials is constrained by a “geometry‐determines‐performance” paradigm, resulting in narrow bandwidth and complex fabrication processes. Here we introduce a chemically programmable metamaterial that overcomes this limitation by shifting the control logic from geometric design to intrinsic material programmability. Through the directed self‐assembly of MXene (Ti_3_C_2_T_x_) nanosheets with polyvinyl alcohol (PVA), we fabricated a core film with a nanoscale quasi‐periodic layered structure, which was subsequently laminated with styrene‐butadiene rubber (SBR). The film integrates stable chemical cross‐links and dynamic hydrogen bonds into an editable chemical‐physical multi‐level constraint system. This architecture enables multidimensional programming capabilities. Chemically, adjusting the crosslinker concentration acts as a “chemical scissor” that broadly and continuously tailors the local resonance bands and dynamic states of the material. This programming operates at a deep subwavelength thickness of merely 10 mm, achieving an average absorption coefficient of 0.90 across the 1000–4000 Hz frequency range. Physically, modulating the film thickness and layer count provides an additional control dimension. This physical tuning strategy enhances low‐frequency performance, elevating the absorption coefficient at 600 Hz to 0.70. This work establishes chemical programming as a paradigm for designing metamaterials, transcending the limitations of conventional geometric approaches.

## Graphical abstract

MXene self‐assembly creates a chemically programmable underwater metamaterial with quasi‐periodic layers and a chemical‐physical multi‐level constraint system. A crosslinker as a ‘chemical scissor’ precisely edits microscopic interactions, exciting deep‐subwavelength strong local resonances that induce near‐zero/negative effective bulk modulus (K_eff_) and critical damping (Re(Z_eff_/Z_0_)→1, Z_eff_: effective impedance, Z_0_: water). This provides a foundational paradigm for next‐generation adaptive underwater acoustic metamaterials.

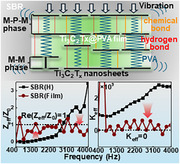

## 1 Introduction

The effective manipulation and absorption of sound waves are crucial for applications in communication, detection, and stealth technologies [1, 2]. Achieving precise acoustic control hinges on the free tailoring of a medium's equivalent constitutive parameters [3, 4]. Acoustic metamaterials are engineered with subwavelength artificial structures, which enable effective constitutive parameters (e.g., negative mass density and negative bulk modulus) that are unattainable in natural materials. These capabilities significantly expand the design freedom for acoustic materials [5, 6, 7, 8]. However, translating this potential into practical technology faces severe challenges, particularly in underwater scenarios.

The acoustic performance of current underwater sound‐absorbing metamaterials relies on pre‐designed, fixed physical structures (e.g., Helmholtz resonators, flexible tube gratings [9, 10, 11, 12], flexible tube gratings [13, 14], phononic crystals [15, 16, 17, 18], membrane‐type structures [19, 20, 21, 22], and composite Metasurfaces [23, 24, 25, 26]). These structures operate by constructing resonant systems that excite a negative or near‐zero effective mass density or bulk modulus. This mechanism enables the manipulation and absorption of sound waves. This geometry‐determines‐performance paradigm results in a narrow acoustic response bandwidth after fabrication [24, 27, 28]. Achieving broadband response necessitates the integration of multiple resonant units [29, 30, 31, 32]. However, this integration approach results in bulky structures and complicated fabrication processes. Furthermore, it poses a significant challenge in the co‐optimization of low‐frequency and broadband performance under ultra‐thin constraints. This paradigm fundamentally limits the intrinsic tunability of the materials. Previous studies have explored performance tuning through deformable structures or external fields [33, 34]. However, challenges still exist. Geometry‑based reconfiguration often relies on complex mechanical structures or deformable components, which inevitably increase system volume and complexity while limiting response speed and reversibility. Externally responsive systems require continuous energy input and external field control devices, which does not meet the demand for compact, passive subwavelength tunability in most application scenarios.

These challenges lead to a pivotal question: can the design of underwater sound‐absorbing metamaterials break away from its singular reliance on, and complex integration of, geometric structures? The answer to this question will dictate their future potential for both scientific research and engineering applications.

To address this, our study proposes a paradigm‐shifting strategy. Leveraging the nano‐layered structure and surface chemistry of two‐dimensional MXene (Ti_3_C_2_T_x_) [35, 36, 37], we constructed an intrinsically programmable underwater sound‐absorbing metamaterial via chemical self‐assembly. In contrast, our “chemical programming” strategy embeds programmability directly into the material's intrinsic structure through molecular‐level chemical bonding regulation, enabling continuous and reversible acoustic modulation at the subwavelength scale without the need for additional mechanical components or external field sources. This material embodies a multi‐level dynamic chemical‐physical multi‐level constraint system. Specifically, using Ti_3_C_2_T_x_ nanosheets as structural building blocks, we achieved self‐assembly with polyvinyl alcohol (PVA) through a glutaraldehyde‐mediated acetalization reaction. This process yielded a Ti_3_C_2_T_x_@PVA film featuring a nanoscale quasi‐periodic layered structure. This film was subsequently laminated with styrene‐butadiene rubber (SBR) to produce the final Ti_3_C_2_T_x_@PVA/SBR composite metamaterial, designated SBR(Film). The chemical cross‐linking network provides a stable skeleton and strong constraints for the system. Concurrently, the dynamic hydrogen‐bonding network imparts adaptive interfacial response capabilities to the material. Together, they synergistically form the core mechanism for property regulation: the chemical‐physical multi‐level constraint system. By simply adjusting the crosslinker dosage, we can precisely chemically program the metamaterial's resonance states. This programming controls its spectrum of near‐zero/negative effective bulk modulus, akin to editing software code. Furthermore, different assembly routes [38, 39]—such as vacuum filtration, spin coating, or interfacial self‐assembly—lead to variations in interlayer spacing, sheet orientation, and defect distribution, which may further influence mechanical and acoustic properties. This is also a direction worth exploring in the future. In summary, this bottom‐up strategy establishes a new paradigm for constructing acoustic metamaterials, replacing macroscopic geometric design and integration with self‐assembly and chemical programming. It thereby opens new avenues for developing next‐generation metamaterials.

## 2 Results and Discussion

### 2.1 Structural Design Strategy and Characterization of Ti_3_C_2_T_x_@PVA Film

**FIGURE 1:**
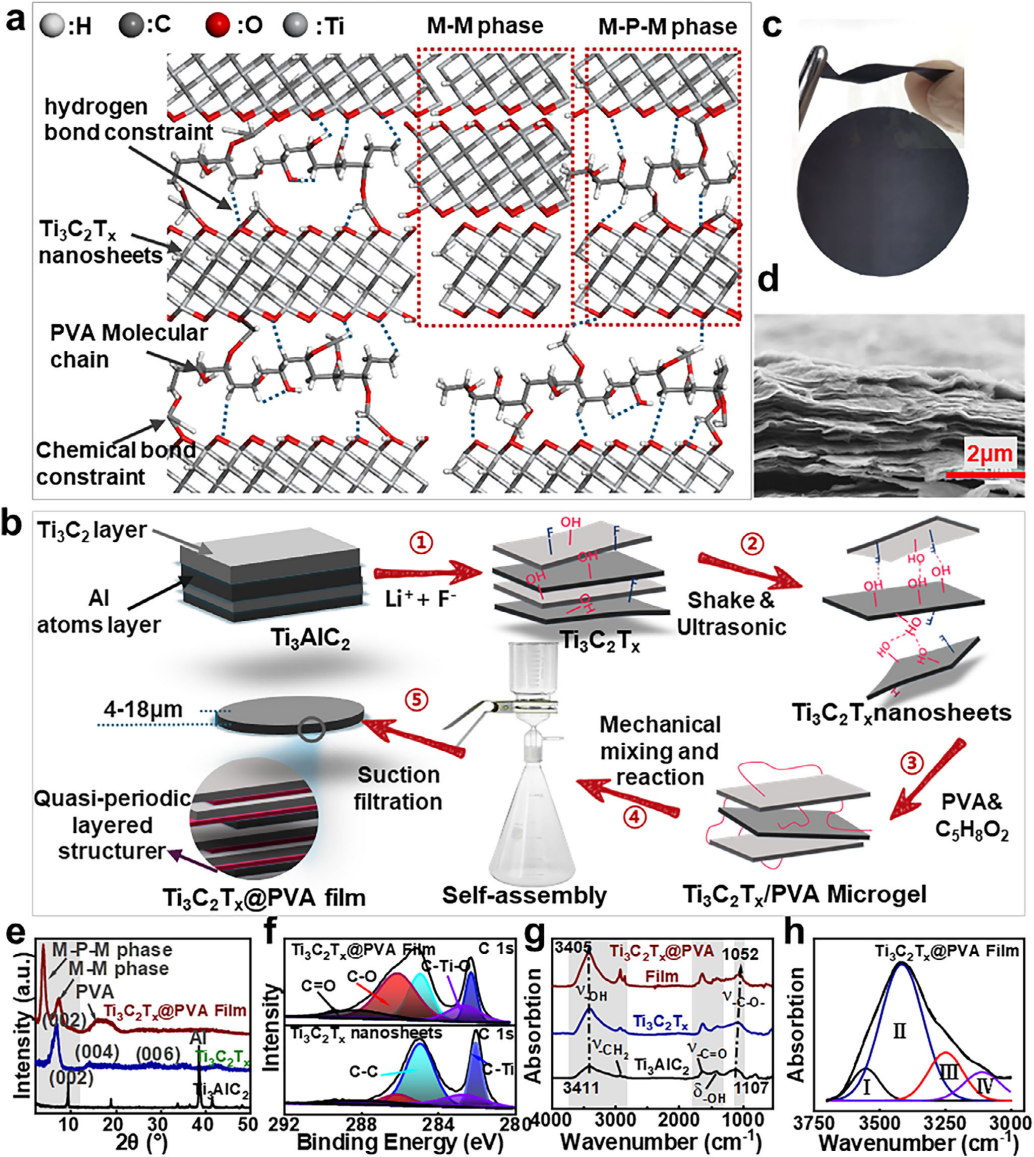
Structural design strategy and characterization of the Ti_3_C_2_T_x_@PVA film. (a) Schematic of the nanoscale quasi‐periodic layered structure, showing phase composition and the chemical‐physical multi‐level constraint system. (b) Flowchart of the self‐assembly process. (c) Digital photograph of the flexible Ti_3_C_2_T_x_@PVA film. (d) Cross‐sectional SEM image revealing the internal nanolaminated morphology. (e) XRD spectra of Ti_3_AlC_2_, Ti_3_C_2_T_x_, and Ti_3_C_2_T_x_@PVA film. (f) XPS C 1s spectra of Ti_3_C_2_T_x_ and Ti_3_C_2_T_x_@PVA film. (g) FTIR spectra of Ti_3_AlC_2_, Ti_3_C_2_T_x_, and Ti_3_C_2_T_x_@PVA film. (h) Spectral deconvolution of the hydrogen‐bonding configurations in Ti_3_C_2_T_x_@PVA film.

To verify the feasibility of replacing traditional macro‐geometric design with chemical programming for constructing acoustic metamaterials, we designed and fabricated a core acoustic component: the Ti_3_C_2_T_x_@PVA film. It possesses a nanoscale quasi‐periodic layered structure consisting of alternating Ti_3_C_2_T_x_/PVA phases (M‐P‐M phase) and closely self‐stacked Ti_3_C_2_T_x_ phases (M‐M phase). A chemical‐physical multi‐level constraint system is introduced between the layers, where a chemically crosslinked network collaborates with a dynamic hydrogen bonding network (Figure 1a). This shifts the performance control logic from physical geometry to the phase composition and interface interactions, enabling intrinsic programming of the underwater sound absorption performance.

The Ti_3_C_2_T_x_@PVA film is obtained through a scalable chemical self‐assembly method (Figure 1b). Ti_3_C_2_T_x_ nanosheets were prepared by selectively etching the Al atomic layers of Ti_3_AlC_2_ and ultrasonically exfoliating the material. These nanosheets were then mixed with PVA and chemically crosslinked with glutaraldehyde to form microgels. During vacuum‐assisted filtration, the microgels undergo hydrogen bonding reconstruction and lamellar stacking, resulting in the fabrication of flexible, micron‐thick Ti_3_C_2_T_x_@PVA films (Figure 1c). Scanning electron microscopy (SEM) images reveal its nanoscale layered structure (Figure 1d).

To confirm the phase composition and the construction of the chemical‐physical multi‐level constraint system within the Ti_3_C_2_T_x_@PVA film, we conducted a comprehensive characterization.

The X‐ray diffraction (XRD) pattern in Figure 1e shows the phase composition of Ti_3_C_2_T_x_@PVA films. After selective etching, the characteristic Al peak at 39.62° in Ti_3_AlC_2_ disappears, and the (002) diffraction peak shifts from 9.4° to 6.73°. These changes confirm the successful synthesis of Ti_3_C_2_T_x_ nanosheets. The (002) peak of Ti_3_C_2_T_x_@PVA film splits into two characteristic peaks at 3.91° and 7.33°, corresponding to interlayer spacings of 4.53 nm for the M‐P‐M phase and 1.21 nm for the M‐M phase. This confirms the successful construction of the desired nanoscale quasi‐periodic layered structure.

The XPS C 1s spectrum provides direct evidence of the chemical constraints (Figure 1f). Compared to pure Ti_3_C_2_T_x_, the characteristic peak for C─O─C bond at 286.1 eV is significantly enhanced in Ti_3_C_2_T_x_@PVA films. Concurrently, the binding energy of C─Ti bonds shifts positively from 282.0 to 282.2 eV, accompanied by an increase in the C─Ti─O bond signal. These findings collectively verify the occurrence of an acetalisation reaction between glutaraldehyde, PVA, and MXene surface hydroxyl groups, successfully establishing a chemical crosslinking network based on acetal bonds.

Fourier‐transform infrared (FTIR) spectroscopy reveals the successful construction of the chemical‐physical multi‐level constraint system in Ti_3_C_2_T_x_@PVA films (Figure 1g). Relative to Ti_3_AlC_2_, the etched Ti_3_C_2_T_x_ displays a markedly intensified –OH absorption at 3410 cm^−1^, confirming the introduction of abundant surface hydroxyls. In the Ti_3_C_2_T_x_@PVA film, the ─C─O─ stretching vibration redshifts from 1107 to 1054 cm^−1^, and the ─C═O band at 1628 cm^−1^ gains intensity, corroborating chemical crosslinking. Simultaneously, the ─OH stretching vibration broadens, intensifies, and redshifts to 3405 cm^−1^, while the bending vibration at 1401 cm^−1^ narrows accordingly. This confirms the presence of a physical hydrogen bonding network. Furthermore, peak fitting analysis identifies four hydrogen‐bonding modes (Figure 1f): free ─OH (I, 3544 cm^−1^), intramolecular self‐associated ─OH (II, 3409 cm^−1^), intramolecular cyclic ─OH (III, 3251 cm^−1^), and intermolecular ─OH···O═C (IV, 3120 cm^−1^). These features collectively confirm a dynamic and diverse physical hydrogen‐bonding network.

In summary, these results collectively indicate that Ti_3_C_2_T_x_@PVA films contain both a stable chemical crosslinking network formed by hemiacetal bonds and a dynamic physical bonding network formed by various types of hydrogen bonds. Together, they construct a chemical‐physical multi‐level constraint system. This lays the material foundation for the chemical programming of underwater sound‐absorbing metamaterials.

### 2.2 Acoustic Response of the SBR(Film)

**FIGURE 2:**
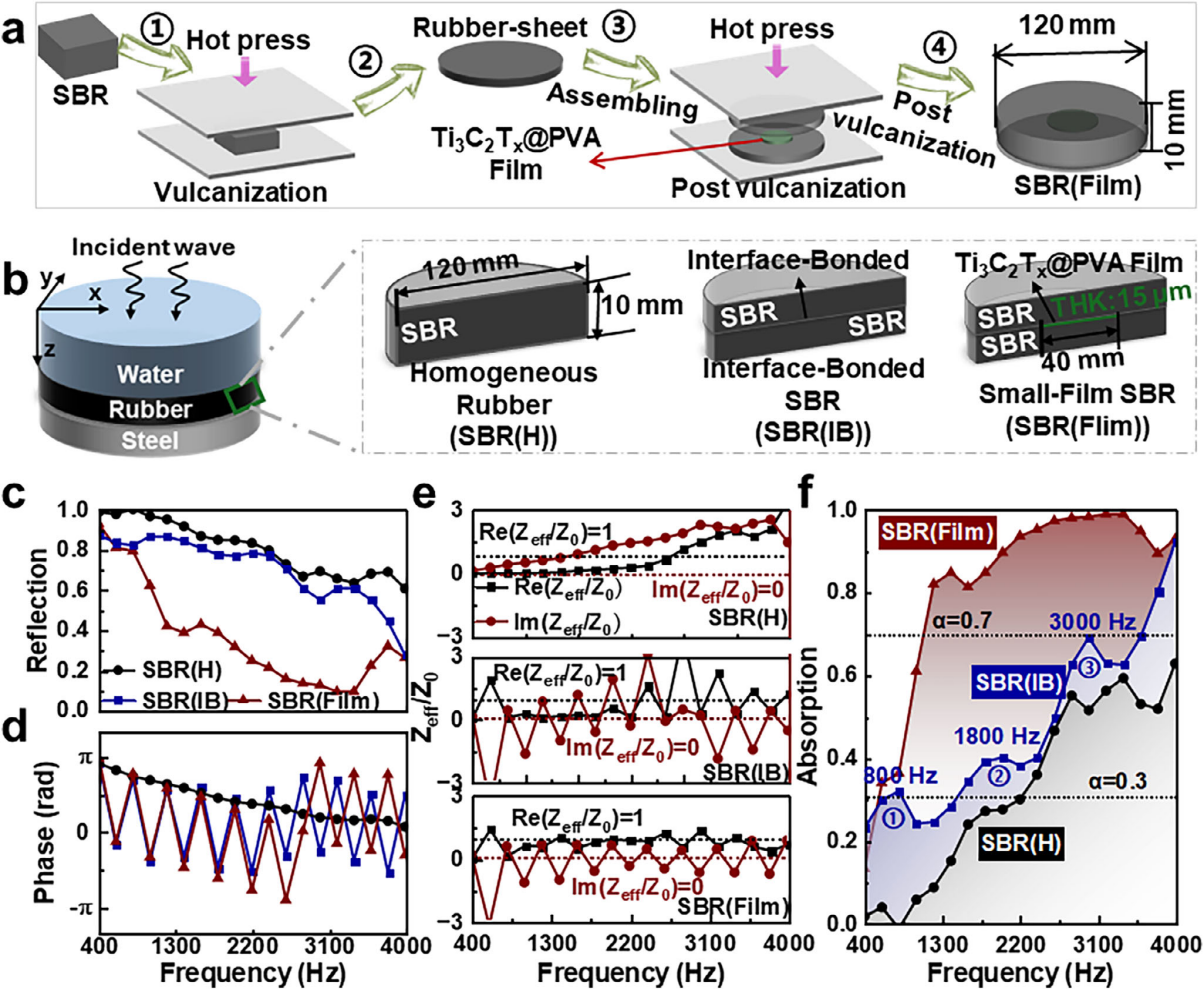
Acoustic response of SBR(H), SBR(IB), and SBR(Film). (a) Fabrication process of the acoustic samples. (b) Schematic diagrams of the test samples and cross‐sectional structure of the rubber components. (c) Reflection coefficients. (d) Reflection phases. (e) Normalized acoustic impedance. (f) Sound absorption coefficients.

To further verify the acoustic effectiveness of the Ti_3_C_2_T_x_@PVA film as a core component, we laminated it with SBR to fabricate an acoustic sample, denoted as SBR(Film) (Figure 2a). We prepared two control samples: homogeneous SBR (SBR(H)) and SBR with basic resonant units formed by vulcanized interfaces (SBR(IB)) (Figure 2b). All samples maintained a deep subwavelength thickness of 10 mm (approximately λ/375 at 400 Hz).

Underwater acoustic tests confirmed that by introducing vulcanized interfaces and Ti_3_C_2_T_x_@PVA films, we successfully achieved intrinsic programming of the material's acoustic response.

First, the reflection coefficient and phase (Figure 2c,d) revealed the evolution of the acoustic energy dissipation mechanism. SBR(H) exhibits typical viscoelastic frequency response characteristics. As the frequency increases, the reflection coefficient gradually decreases from 1.00 to 0.80, and the reflection phase shifts from π to π/2. This indicates that the sound energy loss is primarily attributed to phase shifts and hysteresis induced by the viscoelasticity of the rubber. In contrast, SBR(IB) showed discrete structural resonances induced by the vulcanized interface, superimposed on the viscoelastic response. Reflection “dips” appeared at 800, 1800, and 3000 Hz, accompanied by phase shifts (phase kinks). A more profound transformation was observed with SBR(Film). The introduction of the Ti_3_C_2_T_x_@PVA film extended the discrete resonance points into a continuous broad frequency resonance band (1000–4000 Hz), accompanied by sustained phase kinks. As a result, SBR(Film)’s average reflection coefficient across 400–4000 Hz drops significantly to 0.37, compared to 0.81 for SBR(H) and 0.71 for SBR(IB). This successfully converted the acoustic boundary from highly reflective to highly absorptive.

This transformation is attributed to the precise modulation of the system's acoustic impedance by the Ti_3_C_2_T_x_@PVA film. The normalized acoustic resistance (Re(Z_eff/_Z_0_)) and reactance (Im(Z_eff/_Z_0_)) are shown in Figure 2e. SBR(H) exhibits an inertial response with severe impedance mismatch, as Im(Z_eff/_Z_0_) remains positive and higher than Re(Z_eff/_Z_0_) across the frequency range. While SBR(IB) excites resonances, its Re(Z_eff/_Z_0_) is generally below 1, and Im(Z_eff_/Z_0_) exhibits continuous alternations between positive and negative values with multiple zero‐crossings. This continuous underdamped state limits the effective broadening of the resonance frequency points. In contrast, SBR(Film) demonstrates near‐critical damping resonance state. Across the 400–4000 Hz range, Re(Z_eff_/Z_0_) approaches 1, and Im(Z_eff_/Z_0_) approaches 0. This result indicates near‐ideal impedance matching with water. As a result, sound energy dissipation occurs within a few vibration cycles, broadening the resonance frequency band.

Ultimately, the absorption coefficient spectrum (Figure 2f) shows this performance shift. SBR(IB) exhibits multiple absorption peaks after introducing vulcanized interfaces, SBR(Film) demonstrates superior broadband performance. It achieves an average absorption coefficient of up to 0.90 in the 1000–4000 Hz range, Furthermore, it maintains effective absorption (0.36) in the more challenging 400–1000 Hz low‐frequency range. This indicates that the synergy between the vulcanized interface and the Ti_3_C_2_T_x_@PVA film structure helps overcome the long‐standing challenge of low‐frequency broadband sound absorption at deep sub‐wavelength scales.

### 2.3 Metamaterial Properties of SBR(Film)

**FIGURE 3:**
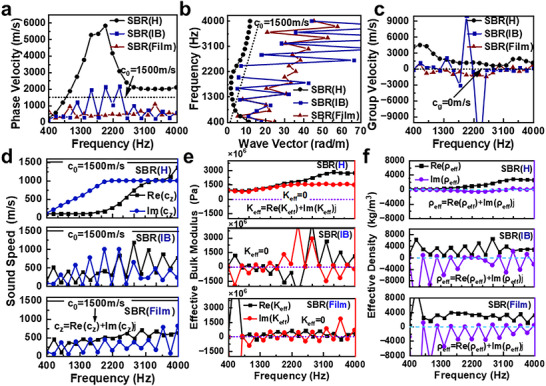
Metamaterial properties of the acoustic samples. (a) Phase velocity, (b) dispersion relation, (c) group velocity, (d) complex sound velocity, (e) effective dynamic bulk modulus (K_eff_), and (f) effective mass density (ρ_eff_) as functions of frequency for SBR(H), SBR(IB), and SBR(Film).

The equivalent dynamic parameters of the composite metamaterial in this study are all derived from measured data obtained using an underwater acoustic impedance tube, retrieved via the one‐dimensional transfer matrix method (TMM) adapted for layered viscoelastic systems(See the Supporting Information for details). At the fundamental physics level, SBR(Film) provides a novel and ideal model system for investigating strong wave‐matter interactions and wide‐frequency anomalous physics. Further analysis was conducted on the acoustic parameters of SBR(H), SBR(IB), and SBR(Film) (Figure 3). The results demonstrate that the sequential introduction of vulcanized interfaces and Ti_3_C_2_T_x_@PVA films synergistically induces a classic slow‐sound effect. This effect significantly reduces the average phase velocity of SBR(Film) to 497 m/s, compared with 2660 m/s for SBR(H) and 731 m/s for SBR(IB) (Figure 3a). This effect is accompanied by dispersive behavior (Figure 3b) and a significant number of near‐zero and negative group velocity frequency points (Figure 3c and Table S1). Meanwhile, the complex sound velocity of SBR(Film) significantly decreased (Figure 3d). This indicates that the resonances in SBR(IB) and SBR(Film) are localised. Sound energy is effectively confined and dissipated within the vulcanized interface and Ti_3_C_2_T_x_@PVA film. This enables acoustic control at deep sub‐wavelength scales.

More importantly, the localized resonances thus triggered led to anomalous frequency responses in the effective bulk modulus (K_eff_) and mass density (ρ_eff_) (Figure 3e,f). Compared to SBR(H), BR(IB) shows multiple near‐zero or negative values in K_eff_ in the 400–2600 Hz range, crossing zero several times. SBR(Film) further extends this tuning range to 400–4000 Hz, with the K_eff_ values approaching zero (Figure 3e). Additionally, the ρ_eff_ of both SBR(IB) and SBR(Film) transitions from a monotonically increasing trend in SBR(H) to a nearly constant value across the entire frequency range (Figure 3f). This ability to achieve ‘wide‐frequency anomalous’ tuning of the material's fundamental parameters is crucial. It enables the realization of multi‐frequency, wideband localized resonances. This ability represents the core goal traditionally pursued by metamaterials through complex macroscopic geometric design.

### 2.4 Chemical Modulation of SBR(Film) Metamaterial Properties

Based on the localized resonance of the Ti_3_C_2_T_x_@PVA film and the vulcanized interface, the properties of the SBR(Film) metamaterial can be continuously, reversibly, and precisely programmed. This can be achieved through the coordinated reconfiguration of the phase composition and the chemical‐physical multi‐level constraint system of Ti_3_C_2_T_x_@PVA film.

**FIGURE 4:**
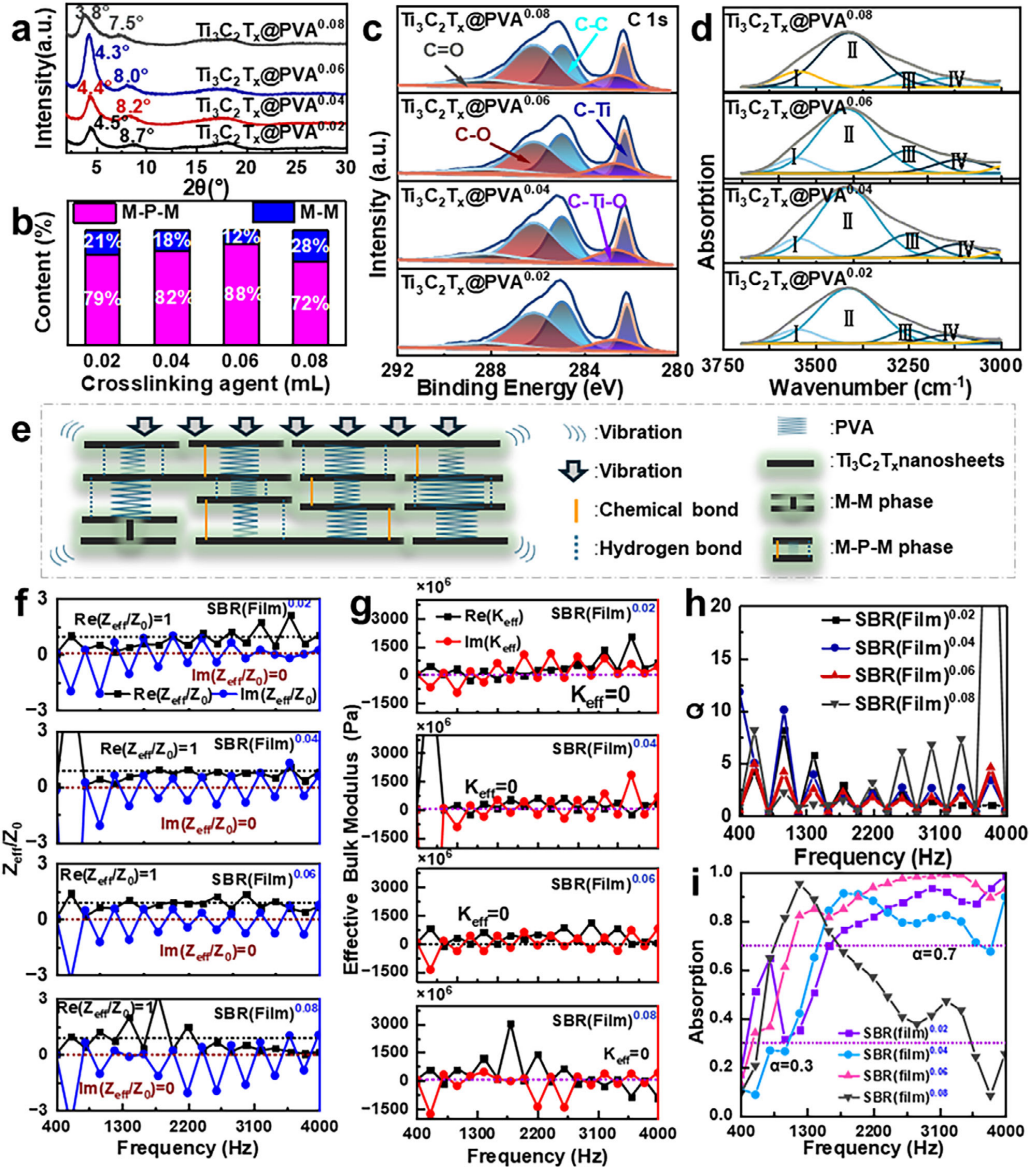
Chemical modulation of the SBR(Film) metamaterial properties. (a) XRD spectra, (b) Phase proportion diagram, (c) XPS C 1s spectra, and (d) evolution of hydrogen‐bonding configurations for the Ti_3_C_2_T_x_@PVA^y^ films. (e) Schematic of the chemical‐physical multi‐level constraint system within the film. (f) Normalized acoustic resistance and reactance, (g) effective dynamic bulk modulus, (h) acoustic quality factor, and (i) sound absorption coefficient for the corresponding SBR(Film)^y^ composites. The variable “y” in Ti_3_C_2_T_x_@PVA^y^ and SBR(Film)^y^ denotes the volume of glutaraldehyde crosslinker added to a mixture of 40 mL of 1.5 mg/mL Ti_3_C_2_T_x_ solution and 4 mL of 0.2 wt% PVA solution.

By controlling the amount of crosslinking agent (denoted as y), we fabricated a series of Ti_3_C_2_T_x_@PVA^y^ films (y = 0.02‐0.08 mL). XRD analysis (Figure 4a) shows that the crosslinker content effectively modulates the phase composition and structural ordering of the layered structure. As the crosslinker amount increases, the interlayer spacing undergoes a slight expansion. The M‐P‐M phase peak shifts from 4.5° to 3.8°, indicating a layer spacing increase from 1.93 to 2.33 nm. Concurrently, the M‐M phase peak shifts from 8.7° to 7.51°, with its spacing increasing from 1.02 to 1.18 nm. At the same time, the proportions of M‐P‐M and M‐M phases show a nonlinear change, first increasing and then decreasing (Figure 4b). The maximum ratio of 7.63:1 occurs at 0.06 mL, where the proportion of the defect phase M‐M is at its lowest and the structural order is at its highest. This indicates that we can use chemical methods to regulate structural defects and optimize the long‐range order of the layered structure.

This structural optimization stems from the direct restructuring of the multi‐level constraint system by the crosslinker. The XPS C 1s spectra (Figure 4c) reveal a significant enhancement of the chemical crosslinking network with increasing crosslinker content. The intensity of the C–O–C peak—a signature of the acetalisation reaction at 286.4 eV—grows markedly and shifts to 286.8 eV. FTIR analysis further reveals the complex evolution of the dynamic hydrogen‐bonding network (Figure 4d). Specifically, the hydrogen bond distribution undergoes a nonlinear transformation with increasing crosslinking agent. The proportions of more ordered intramolecular cyclic (III) and intermolecular (IV) hydrogen bonds both reach a maximum at 0.06 mL (all fitting results in Table S2, standard deviation < 5.00%). This extremum corresponds to a more stable and ordered hydrogen‐bonding network.

The coordination of the phase composition and the chemical‐physical multi‐level constraint system of Ti_3_C_2_T_x_@PVA is shown in Figure 4e. Here, the stable chemical crosslinking network forms the structural framework, while the dynamic hydrogen bonding network imparts adaptive response capability to the interface.

From the perspective of intrinsic sound‐structure interaction mechanism, different structural components in this chemical‐physical multi‐level constraint system undertake differentiated and synergistic acoustic regulation functions:

The C‐O‐C covalent crosslinking network formed via glutaraldehyde‐mediated acetalization reaction acts as the rigid skeleton of the system, which directly determines the storage modulus and elastic response of the material, and further dominates the frequency evolution of the real part of normalized acoustic impedance (Re(Zeff/Z0)). The continuous increase of crosslinking density systematically elevates the system rigidity, driving a continuous transition of the resonant damping state from underdamping at low crosslinking degree to overdamping at high crosslinking degree, which provides a core chemical handle for precise regulation of impedance matching.

The abundant dynamic hydrogen bonds (including intramolecular and intermolecular hydrogen bonds) in the system undergo reversible breakage and reconstruction under acoustic excitation, and the frictional slip between Ti_3_C_2_T_x_ nanosheets in the M‐M phase region generates additional interfacial damping. These two factors together constitute the dominant source of acoustic energy dissipation. At the optimal crosslinking point (y = 0.06), the proportion of ordered hydrogen bonds reaches the peak while the proportion of defective M‐M phase is minimized, where the reversible kinetic process of dynamic hydrogen bonds and interfacial frictional dissipation capacity reach the optimal level, achieving the maximum acoustic energy dissipation efficiency.

Through chemical modulation, frequency‐specific wave control can be achieved, ultimately leading to exceptional chemical programming capabilities for macroscopic acoustic responses. The acoustic impedance spectra in Figure 4f clearly depict the evolution of the resonant dynamics of SBR(Film) ^y^ with crosslinker content.

At low crosslinking (y = 0.02), SBR(Film)^0.02^ exhibits distinct asymmetric damping separation characteristics. Specifically, an underdamped state (Re(Z_eff_/Z_0_) < 1) in the 400–2000 Hz band, and an overdamped state (Re(Z_eff_/Z_0_) > 1) in the 2000–4000 Hz range. This frequency‐dependent damping separation can be directly associated with the phase composition and multi‐level constraint system of Ti_3_C_2_T_x_@PVA film. At this point, the structure contains the highest proportion of the defective M‐M phase and the sparsest chemical and hydrogen‐bonding networks. This specific structural arrangement produces weak interfacial constraints. This results in a non‐uniform damping distribution where vibration modes across different frequency ranges cannot cooperate.

When the crosslinking degree is increased to the optimal value (y = 0.06), the system transitions to an ideal near‐critical damping state. Its Re(Z_eff_/Z_0_) approaches 1 and Im(Z_eff_/Z_0_) approaches 0 across the entire 400–4000 Hz range. In this state, sound waves penetrate the material with minimal reflection. The sound energy is efficiently dissipated as heat via viscous damping with minimal oscillation. This is a key prerequisite for wideband, high‐efficiency sound absorption.

This optimal resonant dynamic state corresponds structurally to the synergistic balance of phase composition and the chemical‐physical multi‐level constraint system.

At the optimal crosslinking point (y = 0.06), the chemical crosslinking network provides appropriate stiffness matching the aqueous environment, making the real part of the system's acoustic impedance highly consistent with the characteristic impedance of water, which solves the serious impedance mismatch between traditional underwater acoustic materials and water. Meanwhile, the dynamic hydrogen bond network and optimized M‐M phase interface provide maximized viscous dissipation, enabling the acoustic energy entering the material to be efficiently converted into heat and dissipated. The precise synergy of the two allows the system to simultaneously meet the two core requirements of underwater sound‐absorbing materials: impedance matching and high‐efficiency energy dissipation, and finally achieves the near‐critical damping resonance state in the full frequency range and excellent broadband sound absorption performance.

When crosslinking is further increased (y = 0.08), the system's damping distribution undergoes a fundamental reconfiguration and state inversion. The original low‐frequency band (400–2000 Hz) transitions to overdamped (Re(Z_eff_/Z_0_) > 1), while the high‐frequency band (2000–4000 Hz) becomes underdamped (Re(Z_eff_/Z_0_) < 1). This non‐monotonic evolution reveals the decisive role of chemical crosslinking in determining the intrinsic damping distribution of the material. It also demonstrates how chemical programming can reconstruct the resonant dynamic state.

Simultaneously, the anomalous response band of K_eff_ also achieves “chemical tailoring” (Figure 4g). The frequency distribution of the near‐zero/negative modulus phenomena shifts systematically with increasing crosslinker content. This systematic shift progresses from concentration in the low‐frequency 400–2000 Hz range (y = 0.02), to spanning the entire measured spectrum (y = 0.06), and finally to focusing on the higher 2000–4000 Hz range (y = 0.08). This indicates that the crosslinker acts like a “chemical scissor”, precisely tailoring the frequency distribution of the local resonances.

At y = 0.06, SBR(Film)^0.06^ achieves significant resonance band broadening and maximized energy dissipation efficiency (lowest average quality factor of 1.59, Figure 4h). It achieves an average sound absorption coefficient of up to 0.90 in the 1000–4000 Hz range. Additionally, it maintains effective absorption in the challenging 400–1000 Hz sub‐kHz range, with an average absorption coefficient of 0.34 (Figure 4i).

Through a simple chemical modulation strategy, we have established a direct bridge from the microscopic chemical environment to macroscopic acoustic performance. This enables active, continuous, and precise programming of metamaterial acoustic responses.

Further underwater long‐term stability studies (Figure S3) confirm that SBR(Film) remains stable in terms of structure, morphology, and acoustic performance, demonstrating good application prospects.

### 2.5 Physical Modulation of SBR(Film) Metamaterial Properties

The chemo‐physical multi‐level constraint system of the Ti_3_C_2_T_x_@PVA film in SBR(Film) provides not only chemical programmability but also the capability to incorporate physical modulation strategies. This creates a multi‐dimensional modulation approach that spans from the microscopic to macroscopic structure.

**FIGURE 5:**
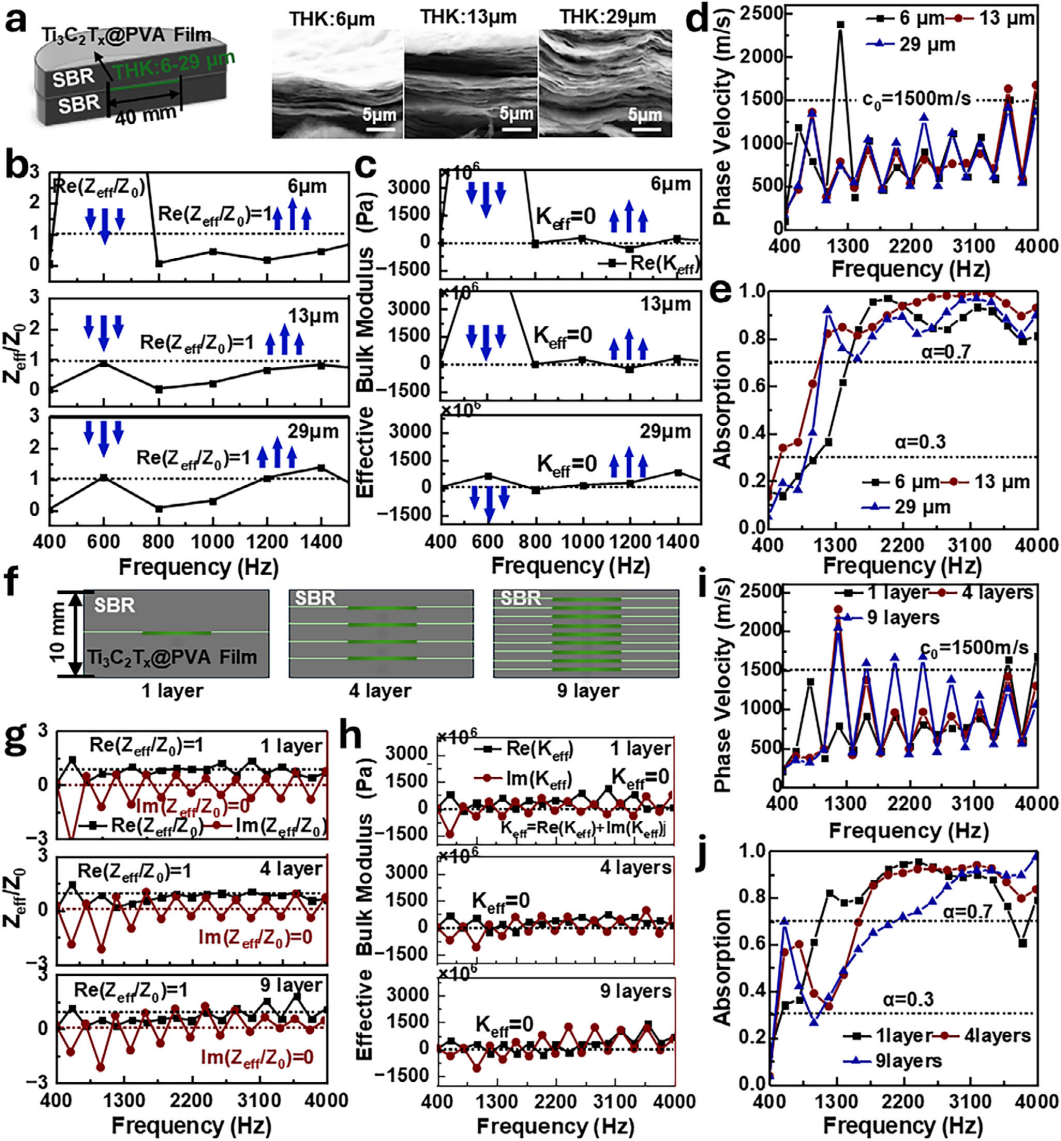
Physical modulation of the SBR(Film) metamaterial properties. (a) Cross‐sectional SEM images of Ti_3_C_2_T_x_@PVA films with different thicknesses. Acoustic properties of SBR(Film) with different Ti_3_C_2_T_x_@PVA film thicknesses: (b) normalized acoustic resistance and reactance, (c) real part of K_eff_, (d) phase velocity, and (e) sound absorption coefficient. Modulation via the number of laminated layers: (f) Schematic of the laminated structure. Acoustic properties of SBR(Film) with different numbers of laminated layers: (g) normalized acoustic resistance and reactance, (h) K_eff_, (i) phase velocity, and (j) sound absorption coefficient. *Note*: Modulation is demonstrated using SBR(Film)^0.06^ as a representative example.

Taking SBR(Film)^0.06^ as an example, we achieved deep subwavelength control over the acoustic properties of SBR(Film) by precisely controlling the thickness of the Ti_3_C_2_T_x_@PVA film (6–29 µm, Figure 5a). As thickness increases, the resonant state of SBR(Film) at specific frequencies undergoes significant transitions. These transitions manifested as a shift from overdamped to critically damped near 600 Hz, and from underdamped to critically damped near 1200 Hz (Figure 5b). The material maintains a near‐critical damping state from 1400 to 4000 Hz (Figure S2a). This phenomenon, where micrometer‐scale thickness variations control sound waves at the meter scale, demonstrates deep sub‐wavelength control over a range of 5–6 orders of magnitude. Further analysis indicates that increased thickness directly modulates local resonance behavior. At 600 Hz, local resonance is activated, and K_eff_ drops from a very high value to nearly zero. Near 1200 Hz, K_eff_ undergoes a fine transition from negative to positive within the near‐zero range (Figure 5c and Figure S2b). This single‐frequency control of K_eff_ arises from the increased layer space and longer propagation paths provided by the thickness increase. This change effectively facilitates interactions between low‐frequency sound waves and more resonance units within the material.

Performance tests confirm this modulation effect. At 13 µm thickness, the phase velocity decreases from 1176.05 to 457.85 m/s at 600 Hz, and from 2374.41 to 724.91 m/s at 1200 Hz, respectively (Figure 5d). The corresponding absorption coefficients increased from 0.14 to 0.34 and from 0.37 to 0.82. Additionally, the 1400–4000 Hz range maintains high sound absorption performance, with an average absorption coefficient of 0.93 (Figure 5e).

By fixing the total thickness at 10 mm, we further optimized the frequency‐selective acoustic performance of SBR(Film) by adjusting the number of laminated layers (*n* = 1, 4, 9; Figure 5f). The lamination design enhances the sub‐kHz frequency acoustic control by introducing multiple Ti_3_C_2_T_x_@PVA films, utilizing interlayer sound field coupling and resonance peak superposition. Unlike thickness control, the laminated structure influences the entire frequency range (Figure 5g). Increasing the layer count drives the sub‐kilohertz range (<1000 Hz) toward the ideal critically damped state, while the high‐frequency range (>1000 Hz) shows asymmetric damping separation. Specifically, an underdamped state (Re(Z_eff_/Z_0_) < 1) emerges in the 1000–3000 Hz range. Concurrently, an overdamped state (Re(Z_eff_/Z_0_) > 1) dominates the 3000–4000 Hz range. Notably, the number of layers primarily alters the system's damping characteristics rather than its resonance frequencies. K_eff_ remains consistently within the near‐zero range (Figure 5h). This behavior demonstrates that its function involves optimizing the energy dissipation path, not altering the intrinsic vibration frequency. Increasing the number of layers also causes a significant decrease in phase velocity in the sub‐kHz range from 1357.75 to 311.99 m/s at 800 Hz (Figure 5i).

The addition of layer control significantly improved the performance in the sub‐kHz range. SBR(Film) with 4 and 9 layers exhibited strong absorption peaks in the sub‐kHz range. The peak absorption coefficients reached 0.60 and 0.70, showing improvements of 66.67% and 105.88% compared to the single‐layer structure (Figure 5j). However, the sound absorption performance at 1000–3000 Hz. These responses reveal an inherent frequency‐response trade‐off in layer number optimization under a fixed total thickness.

Compared to metamaterials relying on precise geometric designs in the literature (Table S3), SBR(Film) achieves denser resonance frequencies, a broader operating bandwidth, and a smaller thickness in the low‐to‐medium frequency range (400–4000 Hz). In contrast, our material achieves this performance through chemical self‐assembly, requiring no complex computations in its preparation process, demonstrating significant advantages.

The synergistic reconfiguration of the phase composition and chemical‐physical multi‐level constraint system in Ti_3_C_2_T_x_@PVA film naturally extends to a chemical‐physical multi‐dimensional modulation strategy. This confirms the feasibility of using chemical programming to replace traditional macroscopic geometric design in the construction of acoustic metamaterials. This enables precise programming of metamaterial properties and lays the foundation for developing the next generation of more designable and versatile metamaterials.

## 3 Conclusion

This work proposes and verifies the feasibility of a fundamental paradigm shift in the design of underwater sound‐absorbing metamaterials: from macroscopic geometric structures to intrinsic chemical programmability.

Through the directed self‐assembly of Ti_3_C_2_T_x_ and PVA, we constructed a core film with a nanostructured quasi‐periodic layered design, which possesses a unique phase composition and a chemo‐physical multi‐level constraint system. When composited with rubber, this material, with just 10 mm of sub‐wavelength thickness, can exhibit anomalous equivalent parameter responses and critical damping resonance in the 400–4000 Hz range, achieving exceptional broadband sound absorption.

Importantly, this paradigm shift allows us to develop a multi‐dimensional strategy for modulating the properties of the metamaterial. Chemically, the concentration of the crosslinking agent acts as a “molecular scissor,” enabling the control of localized resonance bands across the entire frequency range and reversibly driving the system toward an ideal critical damping state. Physically, the adjustment of film thickness and layer count provides complementary modulation, doubling the sound absorption performance in the sub‐kHz frequency range.

This study not only confirms the feasibility of designing chemically programmable underwater sound‐absorbing metamaterials but also addresses the long‐standing challenge of balancing ultra‐thin, low‐frequency, and broadband absorption. More importantly, on the fundamental physics level, it provides an ideal model system for studying complex wave‐matter interactions and broad‐frequency anomalous physics. This approach is anticipated to provide a rational research pathway for the development of the next generation of metamaterials.

## 4 Statistical Analysis

All material characterization results (XRD, XPS, FTIR, SEM) are presented using representative spectra and images to illustrate the typical nanostructure, chemical bonding states, and phase composition of the material. Quantitative peak fitting of the hydrogen‐bonding configurations was performed using the built‐in peak fitting module of Origin.

All acoustic testing and material characterization data in this study were obtained from at least three independent sample preparations and repeated measurements to ensure reliability and reproducibility. Experimental data processing and plotting were conducted using Origin 2023 software. Acoustic parameters were derived from measured data obtained using an underwater acoustic impedance tube and calculated via the one‐dimensional transfer matrix method.

## Author Contributions

**Ziwen Gan**: Writing – review & editing, Methodology, Investigation, Formal analysis, Data curation, Conceptualization. **Ranran Qi**: Writing – review & editing, Validation, Methodology, Formal analysis. **Mingyi Liao**: Writing – review & editing, Supervision, Funding acquisition. **Chen Cheng**: Validation, Methodology, Formal analysis. **Bowen Chen**: Validation. **Wei Tu**: Validation.

## Conflicts of Interest

The authors declare no conflicts of interest.

## Supporting information




**Supporting File**: advs76139‐sup‐0001‐SuppMat.docx.

## Data Availability

The data that support the findings of this study are available from the corresponding author upon reasonable request.
