# Supplementary material for: Chemically Programmable Underwater Sound‐Absorbing Metamaterial via MXene Self‐Assembly
Source: Adv Sci (Weinh). 2026 Jun 15:e76139. Online ahead of print. doi: 10.1002/advs.76139 (PMC13336388; doi:10.1002/advs.76139)
Supplement: Supplementary file 1 — Supporting File: advs76139‐sup‐0001‐SuppMat.docx. [file ADVS-9999-e76139-s001.docx]

**SUPPORTING INFORMATION**

Chemically Programmable Underwater Sound-Absorbing Metamaterials via MXene Self-Assembly

Ziwen Gan, Ranran Qi, Mingyi Liao*, Bowen Chen, Chen Cheng, Wei Tu

College of Transportation Engineering, Dalian Maritime University, Dalian 116026, Liaoning, China

E-mail: [liaomy@dlmu.edu.cn](mailto:liaomy@dlmu.edu.cn)

# Materials and Methods

## Materials

Lithium fluoride (LiF, 98.5%), titanium aluminium carbide (Ti_3_AlC_2_, 400 mesh), polyvinyl alcohol (PVA, Mw=8.9–9.8×10⁴ g/mol), and glutaraldehyde were obtained from Shanghai Macklin Biochemical Technology Co., Ltd.

Hydrochloric acid (HCl, CP, 36–38 wt%), stearic acid (CP), and zinc oxide (CP) were provided by Tianjin Kemiou Chemical Reagent Co., Ltd.

Commercial styrene-butadiene rubber (SBR 1500E) was sourced from PetroChina Co., Ltd. Carbon black was acquired from Cabot Corporation. Sulfur was supplied by Shanghai Aladdin Biochemical Technology Co., Ltd. N-tert-butyl-2-benzothiazole sulfonamide (TBBS) was procured from Ningbo Actmix Rubber Chemicals Co., Ltd.

All materials and chemicals were utilized directly in their as-received state without any additional treatment.

## Preparation of Ti_3_C_2_T_x_ Colloidal Solution

First, at 35°C, a solution was prepared by dissolving 1 g of Ti₃AlC₂ powder in 20 mL of an acidic etching solution (hydrochloric acid and lithium fluoride in a mass ratio of 1:4). This mixture was slowly added to a plastic reactor and etched under magnetic stirring for 48 hours.

Subsequently, the Ti₃C₂Tₓ solution was diluted with water, transferred to centrifuge tubes, and centrifuged at 3500 rpm for 5 minutes to remove the supernatant. This step was repeated until the pH of the supernatant approached 7, yielding a dark green mixture.

Finally, the solution was subjected to ultrasonic treatment for one hour and centrifuged again at 3500 rpm for 5 minutes. The upper layer containing Ti₃C₂Tₓ flakes was collected for subsequent experiments.

## Preparation of Ti₃C₂Tₓ@PVA Films

An aqueous PVA solution with a concentration of 0.2 wt.% was prepared at 60°C. The as-prepared Ti₃C₂Tₓ colloidal solution was then diluted to a concentration of 1.5 mg/mL. Next, 40 mL of the PVA solution was slowly added to 400 mL of the diluted Ti₃C₂Tₓ solution, maintaining a Ti₃C₂Tₓ-to-PVA volume ratio of 10:1. The resulting mixture was sonicated for 5 minutes. Four identical batches of this mixture were prepared, and 0.02, 0.04, 0.06, and 0.08 mL of glutaraldehyde were added to each respective batch, followed by reaction at 60°C for 2 hours to obtain a series of Ti₃C₂Tₓ/PVA microgel solutions. A series of Ti₃C₂Tₓ@PVA films were subsequently obtained via vacuum-assisted filtration.

## Preparation of SBR (Film)

A mixture of 100 g of commercial emulsion-polymerized styrene-butadiene rubber SBR1500E, 50 g of carbon black, 1 g of stearic acid, and 3 g of zinc oxide was compounded on a two-roll mill at 80°C for 30 minutes. After mixing, the compound was passed through the mill twice, then cooled and allowed to rest for 6 hours to ensure a smooth and particle-free surface. Subsequently, 1.75 g of sulfur and 1 g of TBBS accelerator were added and mixed under the same conditions, yielding a fully compounded rubber material.

The curing characteristics of the compound were determined using a rotorless curemeter. The compound was then pre-cured in a platen press to form a 5-mm thick pre-cured rubber sheet, with the pre-curing time set to half of the optimal curing time (t₉₀). A Ti₃C₂Tₓ@PVA film was sandwiched between two such pre-cured rubber sheets and vulcanized again, ultimately yielding the SBR (Film).

# Characterization

The microstructure and morphology of the powders and films were characterized using scanning electron microscopy (SEM, Supera-55-sapphire, Carl Zeiss AG, Germany) and transmission electron microscopy (TEM, JEM-F200, JEOL, Japan).

The phase composition and layered structure of the Ti₃C₂Tₓ@PVA films were analyzed by X-ray diffraction (XRD, D/MAX-Ultima, Rigaku Corporation, Japan) with a Cu target as the radiation source. The scanning range was set from 3° to 50° at a speed of 4°/min.

The surface elemental composition and chemical structure of the samples were examined by X-ray photoelectron spectroscopy (XPS, ESCALAB Xi+, USA). The analysis was performed using an Al Kα X-ray source (1486.6 eV) with an emission power of 250 W. Spectral fitting was conducted using Casa XPS software, and all data were calibrated based on the C1s binding energy at 284.4 eV.

Fourier transform infrared spectroscopy (FT-IR, Spectrum 3, USA) was employed to characterize the overall chemical structure of the Ti₃C₂Tₓ@PVA films. For comparative analysis, 2 mg of the sample was thoroughly mixed with 50 mg of KBr and pressed into a uniform transparent pellet under 10 MPa pressure for 5 minutes. The spectra were collected in the range of 4000–450 cm⁻¹ with 32 cumulative scans and a resolution of 2 cm⁻¹.

The particle size distribution of Ti₃C₂Tₓ was determined using a laser particle size analyzer (LPSA, BT-9300SE, China). The sample was prepared at a concentration of 1.5 mg/mL, and the measurement chamber was rotated at 4000 rpm. Deionized water was added during ultrasonic dispersion. The instrument was set to scan for 180 seconds, repeated twice, with a resolution of 2 seconds.

# Acoustic testing based on a standing wave tube

The underwater acoustic response was measured using an acoustic standing wave tube system^1^, as illustrated in Figure S1a. The tests were conducted in the frequency range of 400–4000 Hz, with the water temperature maintained at 25°C. Prior to testing, the samples were immersed in water for 24 hours. The reflection coefficient (R) was calculated using the following equation:

$$R_{L}=\frac{H_{12}-e^{iks}}{e^{-ikd}-H_{12}}e^{2jkl}$$

where *d* represents the distance between the two hydrophones, and *L* is the distance from the farthest hydrophone to the sample surface. *k* is the wavenumber in water, defined as ω/c, where ω = 2πf and c is the speed of sound in water (1500m/s at 25°C). H_12_ = P_2_/P_1_ represents the transfer function between the sound pressure spectra at positions 1 and 2. Considering the perfectly reflective backing used in the experiment, the sound absorption coefficient α was calculated as follows:

α=1- |R|^2^


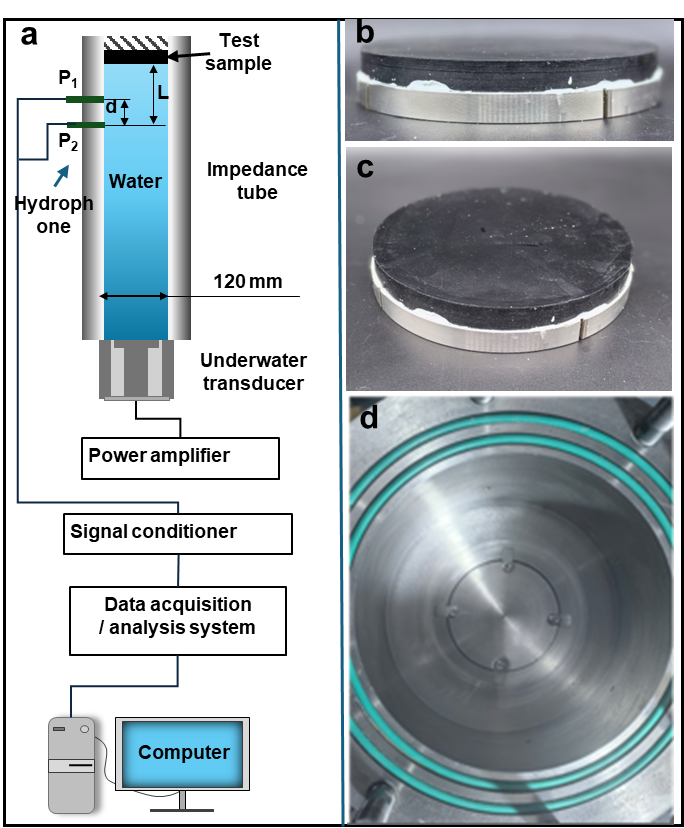


**Figure S1.** (a) Schematic diagram of the water impedance tube. (b) Side view and (c) top view of the SBR(Film) with a rigid backing plate (10 mm thickness), and (d) actual image of the impedance tube sample holder.

# Calculation of Complex Acoustic Impedance at the Interface

Under normal incidence conditions, the relative complex acoustic impedance at the interface of the sound-absorbing material can be calculated based on the reflection phase and reflection coefficient. The calculation formula^2, 3^ is as follows:

$$\frac{Z_{eff}}{Z_{0}}=\frac{1+\left| R \right|e^{\theta i}}{1-\left| R \right|e^{\theta i}}$$

$$\frac{Z_{eff}}{Z_{0}}=Re\left( \frac{Z_{eff}}{Z_{0}} \right)+Im\left( \frac{Z_{eff}}{Z_{0}} \right)i$$

Where $\left| R \right|$ is the reflection coefficient, $\theta$ is the reflection phase, *Z_0_* is the characteristic impedance of water，$Re\left( \frac{Z_{eff}}{Z_{0}} \right)$ is the real part (acoustic resistance), $Im\left( \frac{Z_{eff}}{Z_{0}} \right)$ is the imaginary part (acoustic reactance). Furthermore, the impedance phase (δ) curve can be derived based on Euler's formula.

$$Re\left( \frac{Z_{eff}}{Z_{0}} \right)=\left| \frac{Z_{eff}}{Z_{0}} \right|cos\delta$$

$$Im\left( \frac{Z_{eff}}{Z_{0}} \right)=\left| \frac{Z_{eff}}{Z_{0}} \right|sin\delta$$

# Calculation of Complex Sound Speed

Based on acoustic principles, the solution theory, core equations, and numerical implementation methods for complex sound speed in underwater acoustic standing wave tubes are presented below, which can serve as a standardized reference for experimental data processing and program development. The standing wave tube method is a classic technique for measuring the acoustic properties of materials, whose core lies in deriving the complex sound speed by analyzing the sound pressure standing wave distribution.

Derived from the linear acoustic wave theory and boundary conditions, the core complex equation for solving the complex sound speed is as follows^4, 5^. This equation establishes a quantitative relationship between the composite variable and reflection characteristics:

$$i\cdot\frac{\coth\left( z \right)}{z}=K\cdot\frac{1+Re^{i\varphi}}{1-Re^{i\varphi}}$$

The physical meanings and mathematical expressions of each variable and coefficient in the equation are specified below, among which the complex sound speed and acoustic attenuation coefficient are the core quantities to be solved:

## Composite Variable z

The composite variable z is a key intermediate variable linking attenuation characteristics and propagation characteristics, consisting of a real part (attenuation term) and an imaginary part (propagation term):

$$z=\alpha d+i\cdot\frac{\omega d}{c}$$

## Right-Hand Side Coefficient K

Coefficient K is composed of test system parameters and known physical quantities, reflecting the difference in acoustic properties between water and the measured material:

$$K=\frac{\rho_{w}c_{w}}{\omega\rho d}$$

## Explanation of Physical Parameters

i: Imaginary unit, used to describe the phase relationship in the complex domain

coth: Hyperbolic cotangent function, whose complex form reflects the energy distribution characteristics of the standing wave field

α: Acoustic attenuation coefficient of the material (Np/m) – Quantity to be solved, reflecting the energy loss rate of sound waves in the material

d: Thickness of the material (m), a geometric parameter that needs to be accurately measured in experiments

ω: Angular frequency (rad/s), determined by the frequency of the test signal

c: Complex sound speed of the material (m/s) – Quantity to be solved, whose real part is the phase velocity and imaginary part is related to attenuation

ρ_w_: Density of water (kg/m³), a known quantity approximately 1000 kg/m³ at room temperature

c_w_: Sound speed in water (m/s), a known quantity approximately 1500 m/s at room temperature

ρ: Density of the material (kg/m³), which can be pre-measured by the weight-volume method

R: Amplitude of reflection coefficient (measured value), calculated from the data collected by the standing wave tube pressure sensor array

Φ: Reflection phase (rad), reflecting the phase jump characteristics of sound waves at the material interface

Based on experimental measurement data, the solution of complex sound speed must follow the following standardized steps to realize the conversion from raw data to the terms on both sides of the core equation:

## Calculate the Angular Frequency

Convert the signal frequency set by the test system into the angular frequency:

$$\omega=2\pi f$$

where f is the test frequency (Hz), which should cover the concerned frequency range of the material's acoustic properties.

## Phase Conversion

Convert the reflection phase output by the measurement system from degrees to radians to meet the requirements of trigonometric function calculation:

$$\phi_{\text{rad}} = \phi_{\text{deg}} \times\frac{\pi}{180}$$

## Construct the Complex Reflection Coefficient

Combine the amplitude and phase of the reflection coefficient to construct the reflection coefficient in complex form, which fully describes the reflection characteristics of sound waves:

$$R_{\text{complex}}=R\cdot e^{i\phi_{\text{rad}}}$$

## Calculate the Right-Hand Side Coefficient

Substitute the known physical parameters to calculate the right-hand side coefficient K of the core equation:

$$K=\frac{\rho_{w}c_{w}}{\omega\rho d}$$

## Construct the Right-Hand Side Term

Combine the complex reflection coefficient with coefficient K to complete the construction of the Right-Hand Side (RHS) term of the core equation:

$$RHS=K\cdot\frac{1+R_{\text{complex}}}{1-R_{\text{complex}}}$$

## Define the Composite Variable

Define the composite variable z based on the quantities to be solved (c, α) and known parameters:

$$z=\alpha d+i\frac{\omega d}{c}$$

## Calculate the Left-Hand Side Term

Complete the calculation of the Left-Hand Side (LHS) term of the core equation through the hyperbolic cotangent operation on the composite variable:

$$LHS=i\cdot\frac{\coth\left( z \right)}{z}$$

The core equation is a complex equation, which needs to be decomposed into a system of real equations through separation of real and imaginary parts, and then solved by numerical iteration methods. This method is applicable to the acoustic properties of materials measured by the standing wave tube method.

## Establishment of Equation System

Using the necessary and sufficient condition for complex equality (equal real parts and equal imaginary parts), the core complex equation is decomposed into two coupled real-number equations:

Real part equation:

$$\text{Re}\left( i\cdot\frac{\coth\left( z \right)}{z} \right)=\text{Re}\left( K\cdot\frac{1+Re^{i\phi}}{1-Re^{i\phi}} \right)$$

Imaginary part equation:

$$\text{Im}\left( i\cdot\frac{\coth\left( z \right)}{z} \right)=\text{Im}\left( K\cdot\frac{1+Re^{i\phi}}{1-Re^{i\phi}} \right)$$

## Solution Form

For each test frequency point f, the solution problem is transformed into a root-finding problem of a binary nonlinear equation system, where the variables to be solved are the complex sound speed c and the acoustic attenuation coefficient α:

$$\left\{ \begin{aligned} f_{1}\left( c,a \right)=\text{Im}\left( LHS-RHS \right) \\ f_{2}\left( c,a \right)=\text{Im}\left( LHS-RHS \right) \end{aligned} \right.$$

## Core Numerical Method (Newton-Raphson Method)

For the above nonlinear equation system, the Newton-Raphson method is recommended for iterative solution. This method has a fast convergence speed and is suitable for root-finding problems of smooth functions. The specific steps are as follows:

**Initial Value Setting:** Set the initial values of c and α according to the material type (e.g., refer to the acoustic parameters of similar materials for initial values; the initial sound speed of solid materials can be set to 3000 m/s, and the initial attenuation coefficient to 0.1 Np/m). The rationality of initial values directly affects convergence.

**Jacobian Matrix Construction:** Calculate the partial derivatives of functions f₁ and f₂ with respect to c and α to form the Jacobian matrix J:

$$J=\left[ \begin{matrix} \frac{\partial f_{1}}{\partial c} & \frac{\partial f_{1}}{\partial\alpha} \\ \frac{\partial f_{2}}{\partial c} & \frac{\partial f_{2}}{\partial\alpha} \end{matrix} \right]$$

**Residual Calculation:** Substitute the current iterative values to calculate the residual vector:

$$r=\left[ f_{1}\left( c , \alpha\right) , f_{2}\left( c , \alpha\right) \right]^{T}$$

**Correction Amount Solution:** Solve the linear equation system:

$$Jv=-r$$

to obtain the variable correction vector v.

**Iterative Update:** Update the variable values:

$$\left[ c^{k+1} , \alpha^{k+1} \right]^{T}=\left[ c^{k} , \alpha^{k} \right]^{T}+v$$

where k is the number of iterations.

**Convergence Judgment:** If the 2-norm of the residual is less than the set threshold (e.g. 10^-6^) or the variable update amount is less than the threshold, stop the iteration and output the current value as the solution; otherwise, return to Step 2 to continue the iteration.

To ensure the accuracy and stability of the solution results, the following key points should be focused on during the numerical implementation process. These points are proposed based on the experimental characteristics of standing wave tube measurement and the mathematical characteristics of numerical methods.

## Handling of Nonlinear Characteristics

The strong nonlinearity of the equation originates from the analytical characteristics of the hyperbolic cotangent function coth(z) in the complex domain, whose value changes drastically with the real and imaginary parts of z. During implementation, high-precision numerical computation libraries (such as the coth function in MATLAB or the scipy.special.coth function in Python) should be used to calculate the values of complex functions to avoid the accumulation of truncation errors.

## Selection of Solution Methods

In addition to the Newton-Raphson method, other numerical methods can be selected according to data characteristics:

Least Squares Method: Suitable for scenarios with noise in measurement data, solving the optimal solution by minimizing the sum of squared residuals.

Iterative Transfer Matrix Method: Suitable for multi-sensor standing wave tube systems, improving the solution stability by combining the transfer matrix theory.

## Initial Value Selection Strategy

Initial value selection is the key to the convergence of the Newton-Raphson method. The following strategies are recommended:

For materials with known types, set initial values using pre-experimental data (e.g., the initial sound speed of solid materials is set to 3000 m/s, and the initial attenuation coefficient to 0.1 Np/m).

For materials with unknown types, use the approximate solution in the low-frequency band as the initial value for the high-frequency band (frequency continuity principle) to form an initial value transfer mechanism during frequency scanning.

If iterative divergence occurs, the "small-step perturbation" strategy can be used to adjust the initial values, or the Brouwer-Newton method with more robust convergence can be adopted instead.

## Physical Significance Verification

The solution results must satisfy basic physical constraints; otherwise, they should be determined as invalid solutions and solved again. The core constraint conditions include:

Sound Speed Constraint: c > 0, and it should be within the sound speed range of similar materials.

Attenuation Coefficient Constraint: α > 0 (non-negative energy attenuation).

Frequency Consistency: The sound speed and attenuation coefficient of the same material at adjacent frequency points should change continuously, and abrupt values should be judged in combination with experimental error analysis.

## Environmental Factor Correction

Experimental environmental parameters have a significant impact on the solution results, so a correction link should be added in the numerical implementation:

Temperature Correction: The density and sound speed of water change with temperature (e.g., c_w_ = 1482 m/s at 20℃ and c_w_ = 1507 m/s at 30℃). The corresponding parameters should be substituted according to the experimental temperature.

Boundar

y Correction: Consider the viscous-thermal loss of the standing wave tube wall to correct the measured reflection coefficient, especially in the high-frequency band where the correction effect is more obvious.

# Extraction of Equivalent Dynamic Parameters

**Section S1 Detailed Theoretical Derivation of the One-Dimensional Transfer Matrix Method (TMM)**

This section systematically derives the one-dimensional transfer matrix method (TMM) adapted to the underwater laminated viscoelastic system in this work, which is the core theoretical basis for effective medium parameter extraction and acoustic response calculation. The derivation strictly follows the linear acoustic theory of viscoelastic media, and fully considers the laminated structure characteristics and strong dissipation characteristics of the SBR(Film) metamaterial in this work.

**S1.1 Basic Acoustic Propagation Equations of One-Dimensional Viscoelastic Media**

For underwater acoustic propagation, the incident sound wave is a one-dimensional plane wave propagating along the thickness direction of the sample (defined as the x-axis). For any homogeneous linear viscoelastic medium (including SBR matrix and Ti₃C₂Tₓ@PVA film in this work), the complex dynamic bulk modulus is used to characterize its energy storage and dissipation characteristics, which is defined as:

$\begin{matrix} K(\omega)=K^{'}(\omega)+iK^{''}(\omega) \end{matrix}$ **(S1)**

where $\omega=2\pi f$ is the angular frequency, f is the frequency of the incident sound wave; $K^{'}(\omega)$ is the storage modulus, characterizing the elastic energy storage capacity of the medium; $K^{''}(\omega)$ is the loss modulus, characterizing the viscous energy dissipation capacity of the medium; $i$ is the imaginary unit. The loss factor of the medium is defined as $\eta(\omega)=K^{''}(\omega)/K^{'}(\omega)$, which is regulated by the chemical cross-linking network and dynamic hydrogen bond network in the Ti₃C₂Tₓ@PVA film in this work.

The complex sound velocity $c(\omega)$ and complex wave number $k(\omega)$ of the viscoelastic medium are derived from the complex bulk modulus and the medium density $\rho$:

$c\left( \omega\right)=\sqrt{\frac{K\left( \omega\right)}{\rho}}=c^{'}\left( \omega\right)+ic^{''}\left( \omega\right)$ **(S2)**

$k(\omega)=\frac{\omega}{c(\omega)}=k^{'}(\omega)+ik^{''}(\omega)$ **(S3)**

where $c^{'}(\omega)$ is the real part of the sound velocity, characterizing the propagation speed of the sound wave; $c^{''}(\omega)$ is the imaginary part, characterizing the amplitude attenuation of the sound wave during propagation; $k^{'}(\omega)$ is the phase constant, and $k^{''}(\omega)$ is the attenuation constant.

The characteristic impedance $Z(\omega)$ of the medium, which is the core parameter describing the acoustic matching performance, is defined as:

$\begin{matrix} Z(\omega)=\rho\cdot c(\omega)=Z^{'}(\omega)+iZ^{''}(\omega) \end{matrix}$ **(S4)**

where $Z^{'}(\omega)$ is the acoustic resistance (real part), characterizing the energy dissipation capacity; $Z^{''}(\omega)$ is the acoustic reactance (imaginary part), characterizing the energy storage capacity.

For one-dimensional plane wave propagation in the viscoelastic medium, the acoustic wave equation is:

$\begin{matrix} \frac{\partial^{2}p(x,\omega)}{\partial x^{2}}+k^{2}(\omega)p(x,\omega)=0 \end{matrix}$ **(S5)**

where $p(x,\omega)$ is the sound pressure at position x. The general solution of the equation is the superposition of the forward propagating wave (along the +x direction) and the backward reflected wave (along the -x direction):

$\begin{matrix} p(x,\omega)=p_{i}e^{-ikx}+p_{r}e^{ikx} \end{matrix}$ **(S6)**

where $p_{i}$ is the amplitude of the incident wave, and $p_{r}$ is the amplitude of the reflected wave.

According to the linear acoustic momentum equation, the particle vibration velocity $v(x,\omega)$ is related to the sound pressure gradient:

$\begin{matrix} v(x,\omega)=-\frac{1}{i\omega\rho}\frac{\partial p(x,\omega)}{\partial x}=\frac{1}{Z(\omega)}\left( p_{i}e^{-ikx} - p_{r}e^{ikx} \right) \end{matrix}$ **(S7)**

**S1.2 Transfer Matrix Construction of a Single-Layer Medium**

For a single homogeneous viscoelastic medium layer with thickness $d$, we define the input end (sound wave incident side) as $x=0$, and the output end as $x=d$. The sound pressure and particle vibration velocity at the input end are $p_{1}=p(0)$ and $v_{1}=v(0)$, and those at the output end are $p_{2}=p(d) andv_{2}=v(d)$.

Substitute $x=0 and x=d$ into equations (S6) and (S7) respectively, and eliminate the incident and reflected wave amplitudes $p_{i} andp_{r}$, we can obtain the transfer relationship between the acoustic state quantities at the input and output ends of the single-layer medium, which is described by a 2×2 transfer matrix $\boldsymbol{T}$:

$\begin{matrix} \left[ \begin{aligned} p_{1} \\ v_{1} \end{aligned} \right]=\boldsymbol{T}\left[ \begin{aligned} p_{2} \\ v_{2} \end{aligned} \right] \end{matrix}$ **(S8)**

where the transfer matrix $\boldsymbol{T}$ of the single-layer medium is:

$\begin{matrix} \boldsymbol{T}=\left[ \begin{matrix} \cos(kd) & iZ\sin(kd) \\ \frac{i\sin(kd)}{Z} & \cos(kd) \end{matrix} \right] \end{matrix}$ **(S9)**

The transfer matrix has clear physical significance: it fully characterizes the modulation effect of the medium layer on the amplitude and phase of the sound wave, and the matrix elements are completely determined by the intrinsic acoustic parameters ($Z,k$) and thickness $d$ of the medium. For the Ti₃C₂Tₓ@PVA film in this work, the chemical programming strategy (adjusting the crosslinker concentration) modulates the intrinsic dynamic mechanical parameters of the film, thereby changing the transfer matrix of the film layer, and finally realizing the controllable regulation of the overall acoustic response of the metamaterial.

**S1.3 Total Transfer Matrix and Reflection Coefficient Calculation of Multilayer Laminated Structure**

For the multilayer laminated structure of the SBR(Film) metamaterial in this work, the total transfer matrix of the system is the product of the transfer matrices of each functional layer in the order of sound wave propagation. For a laminated structure composed of $N$ medium layers, the total transfer matrix $\boldsymbol{T}_{\boldsymbol{total}}$ is:

$\begin{matrix} \boldsymbol{T}_{\boldsymbol{total}}=\boldsymbol{T}_{\mathbf{1}}\cdot\boldsymbol{T}_{\mathbf{2}}\cdot\cdots\cdot\boldsymbol{T}_{\boldsymbol{N}}=\left[ \begin{matrix} T_{11} & T_{12} \\ T_{21} & T_{22} \end{matrix} \right] \end{matrix}$ **(S10)**

where $\boldsymbol{T}_{\mathbf{1}}$ is the transfer matrix of the first layer (the layer closest to the water incident medium), and $\boldsymbol{T}_{\boldsymbol{N}}$ is the transfer matrix of the last layer (the layer closest to the rigid backing).

The boundary conditions of the underwater acoustic impedance tube test in this work are strictly defined as follows:

1.Incident medium: deionized water, with characteristic impedance $Z_{0}=\rho_{0}c_{0}, where\rho_{0}=1000\text{ }\text{kg/m}^{3}$ is the density of water, and $c_{0}=1500\text{ m/s}$ is the sound velocity in water at room temperature;

2.Backing condition: rigid steel backing, the particle vibration velocity at the backing interface is 0 (i.e., $v_{N}=0$ at the output end of the Nth layer);

3.The sample is closely bonded to the impedance tube wall, no lateral sound leakage, and only one-dimensional plane wave propagation along the thickness direction is considered.

Under the above boundary conditions, substitute $v_{N}=0$ into equation (S8), the input impedance $Z_{in}$ of the front surface of the sample (the interface between water and the sample) is derived as:

$\begin{matrix} Z_{in}=\frac{p_{1}}{v_{1}}=\frac{T_{11}}{T_{21}} \end{matrix}$ **(S11)**

The complex reflection coefficient $r$ of the sample, which is the ratio of the reflected wave amplitude to the incident wave amplitude at the water-sample interface, is calculated by the input impedance and the water characteristic impedance:

$\begin{matrix} r=\frac{Z_{in}-Z_{0}}{Z_{in}+Z_{0}} \end{matrix}$ **(S12)**

The sound absorption coefficient $\alpha$ of the sample, which is the core performance index in this work, is calculated from the modulus of the reflection coefficient:

$\begin{matrix} \alpha=1-|r|^{2} \end{matrix}$ **(S13)**

This formula is completely consistent with the test principle of the underwater impedance tube in this work, and the test data in the main text are all calculated based on this formula.

**S1.4 Definition of the Layer Structure Adapted to the Samples in This Work**

According to the sample preparation scheme in the main text, the layer structure of the SBR(Film) metamaterial is clearly defined as follows, which is the basis for the construction of the total transfer matrix:

1.Single-layer film sample (n=1): The layer sequence along the sound wave propagation direction is: water → SBR matrix layer 1 (thickness $d_{SBR1}$) → Ti₃C₂Tₓ@PVA functional film layer (thickness $d_{film}$, 6-29 μm) → SBR matrix layer 2 (thickness $d_{SBR2}$) → rigid backing. The total thickness of the sample satisfies $d_{total}=d_{SBR1}+d_{film}+d_{SBR2}=10\text{ mm}$, which is consistent with the deep subwavelength thickness defined in the main text.

2.Multilayer film samples (n=4, 9): The layer sequence is an alternating structure of SBR matrix layer and Ti₃C₂Tₓ@PVA film layer, with a total of $2n+1$ layers (n is the number of film layers). The total thickness of the sample is fixed at 10 mm, and the thickness of each SBR layer is evenly distributed, which is consistent with the layer number regulation scheme in Section 2.5 of the main text.

For the control samples in the main text:

- SBR(H): homogeneous SBR medium with a single layer thickness of 10 mm, the total transfer matrix is the transfer matrix of a single SBR layer;

- SBR(IB): two-layer SBR structure with a vulcanized interface, the total transfer matrix is the product of the transfer matrices of the two SBR layers.

**Section S2 Inversion Algorithm Flow of Effective Medium Parameters**

This section details the inversion algorithm flow of the effective medium parameters based on the TMM model and experimental test data, which is used to extract the equivalent dynamic parameters of the SBR(Film) metamaterial, including the equivalent characteristic impedance $Z_{eff}$, equivalent complex sound velocity $c_{eff}$, equivalent mass density $\rho_{eff}$, and equivalent dynamic bulk modulus $K_{eff}$ in the main text.

**S2.1 Core Theoretical Basis of Inversion**

The core idea of effective medium parameter inversion is: the multilayer laminated SBR(Film) sample is equivalent to a homogeneous medium with a thickness of $d_{total}=10\text{ mm}$ (the total thickness of the sample). For each frequency point, the equivalent homogeneous medium has the same acoustic response (complex reflection coefficient) as the actual multilayer sample under the same boundary conditions. By matching the theoretical reflection coefficient of the equivalent medium with the experimental test reflection coefficient, the effective medium parameters of the sample at each frequency point are inversely solved.

For the equivalent homogeneous medium with thickness $d_{total}$, its transfer matrix $\boldsymbol{T}_{\boldsymbol{eff}}$ is written according to equation (S9):

$\begin{matrix} \boldsymbol{T}_{\boldsymbol{eff}}=\left[ \begin{matrix} \cos\left( k_{eff}d_{total} \right) & iZ_{eff}\sin\left( k_{eff}d_{total} \right) \\ \frac{\mathrm{isin} \left( k_{eff}d_{total} \right)}{Z_{eff}} & \cos\left( k_{eff}d_{total} \right) \end{matrix} \right] \end{matrix}$ **(S14)**

where $k_{eff}=\omega/c_{eff}$ is the equivalent complex wave number, and $Z_{eff}$ is the equivalent complex characteristic impedance, which are the two core parameters to be inverted.

Under the rigid backing boundary condition, the input impedance of the equivalent medium is derived from equations (S11) and (S14):

$\begin{matrix} Z_{in,eff}=-iZ_{eff}\cot(k_{eff}d_{total}) \end{matrix}$ **(S15)**

The theoretical complex reflection coefficient of the equivalent medium is:

$\begin{matrix} r_{theo}(Z_{eff},k_{eff},f)=\frac{Z_{in,eff}-Z_{0}}{Z_{in,eff}+Z_{0}} \end{matrix}$ **(S16)**

The experimental test complex reflection coefficient obtained from the impedance tube test is $r_{test}(f)$, which contains the amplitude and phase information of the reflection coefficient at each frequency point, corresponding to the test data in Figure 2c and 2d of the main text. The core of the inversion algorithm is to solve the optimal $Z_{eff}(f)$ and $k_{eff}(f)$ at each frequency point, so that $r_{theo}$ is infinitely close to $r_{test}$.

After obtaining the optimal $Z_{eff}(f)$ and $k_{eff}(f)$, the other effective medium parameters are calculated according to the acoustic constitutive relation:

$\begin{matrix} c_{eff}(f)=\frac{\omega}{k_{eff}(f)}=\frac{2\pi f}{k_{eff}(f)} \end{matrix}$ **(S17)**

$\begin{matrix} \rho_{eff}(f)=\frac{Z_{eff}(f)}{c_{eff}(f)} \end{matrix}$ **(S18)**

$\begin{matrix} K_{eff}(f)=\rho_{eff}(f)\cdot c_{eff}(f)^{2}=Z_{eff}(f)\cdot c_{eff}(f) \end{matrix}$ **(S19)**

These parameters are the core data supporting the metamaterial interpretation in the main text, including the near-zero/negative equivalent bulk modulus, slow sound effect, and impedance matching characteristics.

**S2.2 Detailed Inversion Steps and Algorithm Flow**

The inversion algorithm is implemented based on the nonlinear least squares method, and the inversion is performed independently for each frequency point in the 400-4000 Hz test frequency band, which ensures the accuracy of the frequency dispersion characteristics of the effective parameters. The detailed steps are as follows:

Step 1: Experimental Data Preprocessing

- Obtain the complex reflection coefficient $r_{test}(f)$ of the sample at each frequency point (frequency interval: 10 Hz) from the underwater impedance tube test, including the modulus $|r_{test}(f)|$ and phase $\varphi_{test}(f)$;

- Correct the test data according to the impedance tube calibration standard, eliminate the system error caused by the tube wall attenuation and sensor position deviation, and ensure the accuracy of the input data of the inversion algorithm.

Step 2: Construction of the Objective Function

The objective function $F$ for inversion is constructed as the sum of squared errors between the theoretical reflection coefficient and the experimental test reflection coefficient:

$\begin{matrix} F(Z_{eff},k_{eff})=\left| \text{Re}(r_{theo})-\text{Re}(r_{test}) \right|^{2}+\left| \text{Im}(r_{theo})-\text{Im}(r_{test}) \right|^{2} \end{matrix}$ **(S20)**

where $\text{Re}(\cdot)$ is the real part of the complex number, and $\text{Im}(\cdot)$ is the imaginary part. The inversion goal is to find the optimal $Z_{eff}$ and $k_{eff}$ that minimize the objective function $F$.

Step 3: Initial Value Setting and Iterative Solution

- Set the initial values of the inversion parameters: the initial value of $Z_{eff}$ is the characteristic impedance of the SBR matrix, and the initial value of $k_{eff}$ is the wave number of the SBR matrix, which ensures that the iterative solution can converge quickly and avoid falling into the local optimal solution;

- Use the Levenberg-Marquardt nonlinear least squares algorithm for iterative solution, which is suitable for the nonlinear fitting problem of complex functions and has high convergence accuracy and stability;

- The algorithm is implemented in MATLAB R2023b, and the built-in `lsqnonlin` function is used for iterative calculation.

Step 4: Convergence Judgment

Set the convergence threshold of the objective function to ${10}^{-6}$. When the change of the objective function $F$ between two adjacent iterations is less than the threshold, the iteration is stopped, and the optimal $Z_{eff}$ and $k_{eff}$ at the current frequency point are output. If the iteration does not converge after 1000 iterations, the initial value is adjusted and the iteration is repeated to ensure the reliability of the inversion results.

Step 5: Calculation of Effective Medium Parameters

According to the converged optimal $Z_{eff}(f)$ and $k_{eff}(f)$, calculate the equivalent complex sound velocity $c_{eff}(f)$, equivalent mass density $\rho_{eff}(f)$, and equivalent dynamic bulk modulus $K_{eff}(f)$ at each frequency point according to equations (S17)-(S19), which are the data used in Figure 2e, Figure 5c and other figures in the main text.

Second, the TMM model is based on four core assumptions:

1. **Assumption of normal incidence plane waves**. All underwater acoustic tests in this study strictly adhere to the national standard GB/T 14369-2011 for acoustic performance testing of underwater acoustic materials. The test frequency range (400–4000 Hz) falls entirely within the plane-wave cutoff frequency of the acoustic tube, exciting only normally incident plane waves propagating along the thickness direction of the sample, with no oblique incidence or lateral scattering. This assumption is valid.
2. **Assumption of homogenization for one-dimensional layered media.** For the laminated structure in this study: 1) At the macroscopic level, the SBR matrix and Ti₃C₂Tₓ@PVA films are alternately stacked along the thickness direction, exhibiting in-plane uniformity and isotropy, which allows simplification as a one-dimensional multilayer medium; 2) At the nanoscale, the interlayer spacing (1–5 nm) of the quasi-periodic layered structure within the Ti₃C₂Tₓ@PVA film is nine orders of magnitude smaller than the acoustic wavelength in the test frequency range (0.375–3.75 m). This results in no scattering effects on the acoustic scale, allowing the film to be treated as an effective homogeneous medium. Characterization results from XRD and SEM have validated this structural feature, supporting the validity of this assumption.
3. **Assumption of linear viscoelasticity.** Given the strong dissipative characteristics of this study, under the test sound pressure levels, both the SBR matrix and the Ti₃C₂Tₓ@PVA film remain within the linear viscoelastic regime. Their storage and loss moduli do not vary with the incident sound pressure amplitude, and the complex equivalent parameters can fully describe the energy dissipation behavior synergistically regulated by the chemically crosslinked network and the dynamic hydrogen-bond network. This assumption is valid.
4. **Assumption of ideal interfacial bonding.** Through a two-stage vulcanization process, this study achieves effective bonding between the Ti₃C₂Tₓ@PVA film and the SBR matrix, with no interfacial gaps or delamination. This ensures the continuity of acoustic displacement and acoustic stress across layers, satisfying the interlayer boundary conditions required by the TMM model. This assumption is valid.

Next, regarding the applicability of the model, the specifics are as follows:

1. **Frequency applicability.** The effective frequency range of the model is 400–4000 Hz. Within this range, the total sample thickness (10 mm) remains deeply subwavelength (λ/37.5–λ/375), satisfying the homogenization conditions for effective media.
2. **Structural applicability.** The model is applicable to the “viscoelastic matrix–functional film” laminated structure designed in this study and can accommodate sample configurations with different film thicknesses and layer counts. The chemical programming strategy used in this study only modulates the intrinsic dynamic mechanical parameters of the film without altering the one-dimensional layered structural characteristics. Therefore, the model can fully capture the changes in equivalent parameters induced by variations in crosslinker concentration. If three-dimensional geometrically complex resonant units are introduced, the one-dimensional TMM model is no longer applicable.
3. **Applicability boundaries for dissipative characteristics.** The model fully accounts for the linear viscoelastic dissipation of the system through complex equivalent parameters. It is suitable for the energy dissipation process dominated by the synergistic effects of chemical crosslinking, hydrogen bonding, and local resonance as studied here. The model is fully applicable in conventional underwater acoustic engineering scenarios with low acoustic intensity. However, when the incident sound intensity is so high that the material enters the nonlinear viscoelastic regime, the linear assumption no longer holds, and the applicability of the model diminishes.

# Metamaterial properties of SBR(film)

**Table S1**. Group velocities of SBR(H), SBR(IB), and SBR(film).

| **Frequency[Hz]** | **SBR(H) Group velocity[m/s]** | **SBR(IB) Group velocity[m/s]** | **SBR(Film) Group velocity[m/s]** |
| --- | --- | --- | --- |
| 400 | 4039.83063 | -179.16632 | -64.7046 |
| 600 | 4479.23419 | 1092.55912 | 864.2161 |
| 800 | 4240.27242 | 240.05589 | -856.733 |
| 1000 | 2847.03378 | 375.95244 | -685.343 |
| 1200 | 1916.92693 | -290.52195 | -283.078 |
| 1400 | 1724.46995 | 2088.67766 | -266.519 |
| 1600 | 1351.56327 | -251.94976 | -1066.16 |
| 1800 | 1091.14246 | -3160.01239 | -546.178 |
| 2000 | 1118.49413 | 9942.91959 | -1163.27 |
| 2200 | 1180.76255 | -542.25746 | -1216.98 |
| 2400 | 1067.27808 | -17091.15084 | -1151.14 |
| 2600 | 823.70392 | -234.79555 | -1460.7 |
| 2800 | 598.48829 | -186.70477 | -749.559 |
| 3000 | 611.89126 | -241.71069 | 733.3029 |
| 3200 | 654.80656 | -106.06899 | -296.311 |
| 3400 | 1812.44769 | -338.02921 | 277.4844 |
| 3600 | 1944.62596 | 193.08757 | -490.394 |
| 3800 | 1448.16708 | -308.80179 | -169.903 |
| 4000 | 1118.32313 | 159.76202 | 660.0682 |

**Table S2.** Frequency ranges of negative/near-zero equivalent bulk modulus and material thicknesses of different underwater acoustic absorbing metamaterials.

| **No.** | **Thickness** **[mm]** | **Name** | **Frequency Range and bandwidth with Negative/Near-Zero Equivalent Bulk Modulus** | **ref.** |
| --- | --- | --- | --- | --- |
| **1** | **118** | **Locally multi-resonant metamaterials^6^** | [500-2000 Hz]; bandwidth:1500 | *Appl Acoust* **2021,** *172*. |
| **2** | **60** | **Material-structure-functionality collaborative optimization metamaterials^7^** | [2500-4000 Hz]; bandwidth:1500 | *International Journal of Mechanical Sciences* **2024,** *281*. |
| **3** |  | **Metamaterials with** **multi-resonator^8^** | [1200-2700 Hz]; bandwidth:1500 | *Appl Acoust* **2019,** *153*, 147-156. |
|  | **53** | **Reconfigurable modular acoustic metamaterial^9^** | [600-2000 Hz]; bandwidth:1400 | *Mech Syst Signal Pr* ***2025,*** *226.* |
| **7** | **50.2** | **Pressure-adaptive ultra-thin hybrid metamaterials^10^** | [358-870 Hz]; bandwidth:512 | *Thin-Walled Structures* **2025,** *216*. |
| **8** | **50** | **Metamaterials with multiple coupling substructure^11^** | [1000-3000 Hz]; bandwidth:2000 | *Journal of Low Frequency Noise Vibration and Active Control* **2025**. |
| **9** |  | **Metamaterials with multilayered locally resonant scatterers^12^** | [3000-3500Hz]; bandwidth:500 | *Results Phys* **2019,** *12*, 132-142. |
| **10** | **45.2** | **Membrane-type acoustic metamaterials^13^** | [300-800Hz]; bandwidth:500 | *Appl Acoust* **2023,** *214*. |
|  | **34.2** | **Meta-structure with multi-order resonance^14^** | [1500-3200Hz]; bandwidth:1700 | *Appl Acoust* ***2021,*** *179.* |
|  | **25** | **Locally resonant piezoelectric metamaterials^15^** | [250-350Hz]; bandwidth:100 | *J Sound Vib* ***2023,*** *548.* |
| **11** | **21.2** | **Hybrid metamaterials using dissipative slow-sound^16^** | [130-180Hz]; bandwidth:50 | *Physics Letters A* **2022,** *450*. |
| **12** | **14** | **Acoustic metamaterial with a mass-in-mass system^17^** | [1150-1200Hz]; bandwidth:50 | *Mechanical Engineering Journal* **2014,** *1* (4) |
| **14** | **10** | **SBR(Film)** | **[1000-4000Hz]; bandwidth:3000** | **this work** |

# Chemical modulation of SBR(Film) metamaterial properties.

**Table 3. Fitting results of -OH hydrogen bonding in Ti_3_C_2_T_x_/PVA hydrogels with varying glutaraldehyde** **dosage.**

| **Ti_3_C_2_T_x_@PVA film** | **Hydrogen bond type** | | **Wavenumber [cm^−1^]** | **Peak area** | **Relative content [%]** | **Standard deviation** |
| --- | --- | --- | --- | --- | --- | --- |
| Ti_3_C_2_T_x_@PVA^0.02^ | Free OH | Ⅰ | 3546.95 | 2.01 | 11.61 | 0.01 |
|  | Self-associated OH | Ⅱ | 3409.47 | 12.09 | 70.10 | 0.02 |
|  | OH…O | Ⅲ | 3249.52 | 1.81 | 10.49 | 0.01 |
|  | Cyclic OH | Ⅳ | 3142.60 | 1.36 | 7.81 | 0.01 |
| Ti_3_C_2_T_x_@PVA^0.04^ | Free OH | Ⅰ | 3546.10 | 2.66 | 11.41 | 0.02 |
|  | Self-associated OH | Ⅱ | 3413.54 | 14.66 | 63.07 | 0.04 |
|  | OH…O | Ⅲ | 3248.00 | 3.86 | 16.60 | 0.04 |
|  | Cyclic OH | Ⅳ | 3113.07 | 2.14 | 8.91 | 0.03 |
| Ti_3_C_2_T_x_@PVA^0.06^ | Free OH | Ⅰ | 3552.31 | 2.06 | 9.37 | 0.02 |
|  | Self-associated OH | Ⅱ | 3414.44 | 13.70 | 62.30 | 0.03 |
|  | OH…O | Ⅲ | 3247.68 | 3.91 | 17.81 | 0.03 |
|  | Cyclic OH | Ⅳ | 3108.52 | 2.46 | 10.53 | 0.03 |
| Ti_3_C_2_T_x_@PVA^0.08^ | Free OH | Ⅰ | 3544.39 | 2.32 | 13.16 | 0.03 |
|  | Self-associated OH | Ⅱ | 3408.82 | 11.46 | 65.19 | 0.04 |
|  | OH…O | Ⅲ | 3251.71 | 2.15 | 12.21 | 0.03 |
|  | Cyclic OH | Ⅳ | 3130.44 | 1.71 | 9.44 | 0.02 |


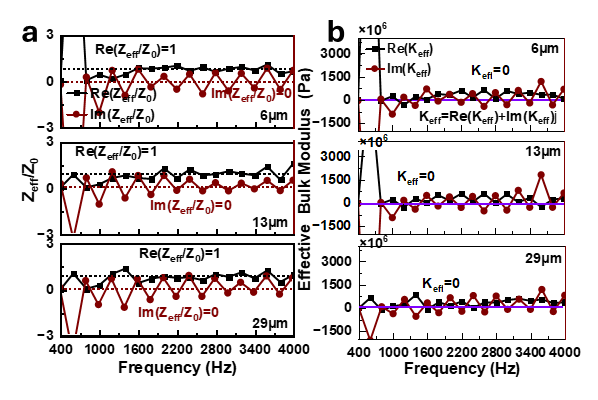


**Figure S2.** (a) Acoustic impedance mappings of SBR(film) across the complete tested frequency range when Ti_3_C_2_T_x_@PVA films with different thicknesses serve as acoustic structural units; (b) Keff spectra of SBR(film) across the complete tested frequency range when Ti_3_C_2_T_x_ @PVA films with different thicknesses serve as acoustic structural units.

# underwater long-term stability studies.

**The outer SBR cladding fundamentally provides a core protective barrier for underwater stability.**

The material in this study adopts an integrated laminated design in which the Ti₃C₂Tₓ@PVA functional film is completely encapsulated by the outer SBR layer. The internal functional film has no direct contact with the external water environment. SBR is an elastomer widely used for sealing in underwater engineering, with low permeability coefficients to water molecules, dissolved oxygen, and salt ions. This physically prevents direct impact of the water environment on the internal functional film and reduces the risk of MXene oxidation. This is one of the core structural advantages that distinguishes our material from other MXene-based underwater acoustic absorption materials.

**We have supplemented underwater immersion experiments to verify the service stability of the material.**

The SBR (Film) 0.06 sample was selected for a 40-day static immersion test in simulated seawater at room temperature. The underwater acoustic absorption performance was measured after 10, 20, and 40 days of immersion. The results show that after 40 days of immersion, the average acoustic absorption coefficient of the sample in the 1000–4000 Hz frequency band remained above 0.82, with a decay rate of less than 10% compared to the initial performance; in the low-frequency band of 400–1000 Hz, the decay rate of the average acoustic absorption coefficient was less than 10%. After immersion, the material still exhibited excellent broadband acoustic absorption performance.

Macroscopic acoustic performance is the most direct macroscopic manifestation of the internal microstructure, chemical crosslinking state, and MXene phase state of the material. Therefore, we infer that if significant MXene oxidation had occurred in the internal functional film, it would inevitably lead to irreversible damage to the dynamic mechanical parameters and local resonance state of the material, ultimately reflected as a substantial decline in acoustic absorption performance. Thus, the core functional structure inside the material can remain stable.


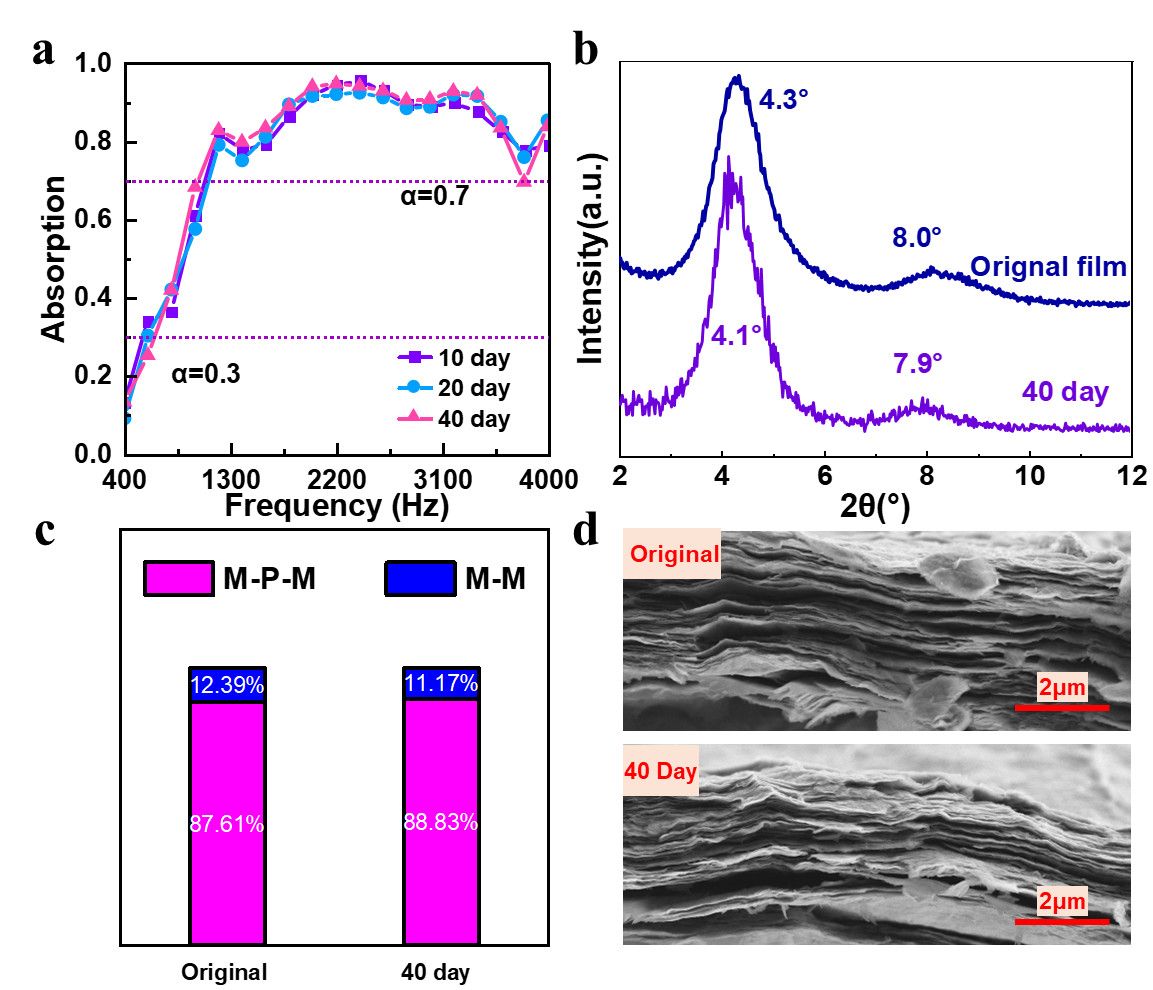


**Figure S3. underwater long-term stability studies.** (a) Sound absorption coefficients. (b) XRD spectra, (c) Phase proportion diagram, (d) SEM spectra.

**The immersed samples were disassembled, and the morphology and structure of the functional film were characterized.**

After immersion, the samples were disassembled for film characterization. SEM results show no significant changes in the surface morphology or layered structure of the film. XRD results show no significant change in the phase structure of the film. The morphology and structure of the functional film exhibit a certain stability during service.

**REFERENCES**

1. Calibration Specification for Acoustic Performance Parameters of Water-Acoustic Materials (Standing Wave Tube Method). JJF 2144-2024.

2. Standard Test Method for Impedance and Absorption of Acoustical Materials Using a Tube, Two Microphones and a Digital Frequency Analysis System. ASTM E1050-19.

3. Acoustics-Determination of sound absorption coefficient and impedance in impedance tubes-Part 2: Transfer-function method. ISO 10534-2:1998.

4. Li, S.; Yi, Y.; Mo, X.; Liu, Y.; He, T.; Tong, H., Standing wave tube measurement method for acoustic and vibration characteristics of underwater acoustic material. *Acta Metrologica Sinica* **2003,** (003), 024.

5. Acoustics-Measurements of the longitudinal wave velocity and attenuation coefficient for underwater acoustical materials-Pulse tube method. GB/T 5266-2006.

6. Gu, Y.; Zhong, H.; Bao, B.; Wang, Q.; Wu, J., Experimental investigation of underwater locally multi-resonant metamaterials under high hydrostatic pressure for low frequency sound absorption. *Appl. Acoust.* **2021,** *172*.

7. Wang, H.; Cui, Z.; He, X.; Ren, Z.; Xiang, P.; Dong, H. W., Underwater acoustic absorbing metamaterials by material-structure-functionality collaborative optimization. *Int. J. Mech. Sci.* **2024,** *281*.

8. Shi, K.; Jin, G.; Ye, T.; Zhang, Y.; Chen, M.; Xue, Y., Underwater sound absorption characteristics of metamaterials with steel plate backing. *Appl. Acoust.* **2019,** *153*, 147-156.

9. Mei, Z.; Shi, T.; Lyu, Y.; Li, X.; Cheng, X.; Yang, J., Reconfigurable modular acoustic metamaterial for broadband sound absorption. *Mech. Syst. Signal. Pr.* **2025,** *226*.

10. Cai, C.; Xin, F., Pressure-adaptive ultra-thin hybrid metamaterials for broadband low-frequency underwater sound absorption. *Thin-Walled Struct.* **2025,** *216*.

11. Shi, K.; Li, D.; Hu, D.; Yu, X.; Ding, C.; Jin, G., Underwater sound absorption characteristics of the acoustic metamaterials with multiple coupling substructure. *J. Low Freq. Noise V. A.* **2025**.

12. Shi, K.; Jin, G.; Liu, R.; Ye, T.; Xue, Y., Underwater sound absorption performance of acoustic metamaterials with multilayered locally resonant scatterers. *Results Phys.* **2019,** *12*, 132-142.

13. Xu, Y.; Hong, Y.; Li, M.; He, X., Underwater low-frequency sound absorption performance and broadband absorption design of membrane-type acoustic metamaterials. *Appl. Acoust.* **2023,** *214*.

14. Wang, L. B.; Ma, C. Z.; Wu, J. H., A thin meta-structure with multi-order resonance for underwater broadband sound absorption in low frequency. *Appl. Acoust.* **2021,** *179*.

15. Wang, M.; Yi, K.; Zhu, R., Tunable underwater low-frequency sound absorption via locally resonant piezoelectric metamaterials. *J. Sound Vib.* **2023,** *548*.

16. Zhou, X.; Duan, M.; Xin, F., Low-frequency underwater sound absorption of hybrid metamaterials using dissipative slow-sound. *Phys. Lett. A* **2022,** *450*.

17. Lu, L.; Otomori, M.; Yamada, T.; Yamamoto, T.; Izui, K.; Nishiwaki, S., Topology optimization of acoustic metamaterials with negative mass density using a level set-based method. *J. Mech. Eng.* **2014,** *1* (4), 1-18.
